# Dose-dependent cardiac autonomic dysregulation under classroom crowding: heart rate variability analysis and machine learning classification

**DOI:** 10.1186/s40101-026-00451-9

**Published:** 2026-10-17

**Authors:** Mohamed Naeem

**Affiliations:** Textile Department, Faculty of Technology and Education, Suez University, P.O. Box: 43221, Suez, Egypt

**Keywords:** Heart rate variability, Classroom density, Cardiac autonomic function, Parasympathetic withdrawal, Machine learning, RoBoSS-SVM, Educational environment, Polyvagal theory, Physiological stress, Wearable monitoring

## Abstract

Classroom overcrowding affects hundreds of millions of students globally, yet its physiological impact on cardiac autonomic function remains uncharacterized. This controlled within-subjects experiment enrolled 30 healthy university students who attended standardized 30-min lectures under three calibrated density conditions: low (30 students/150 m^2^), medium (60/150 m^2^), and high (100/150 m^2^), with all environmental parameters held constant. Twenty-five heart rate variability (HRV) parameters spanning time-domain, frequency-domain, and nonlinear domains were extracted from continuous electrocardiogram (ECG) recordings. Subjective workload was assessed via the NASA Task Load Index (NASA-TLX), and RoBoSS-SVM (a Support Vector Machine using the Robust, Bounded, Sparse, and Smooth loss function) with Recursive Feature Elimination (RFE) classified density conditions from HRV features. Repeated-measures ANOVA revealed significant density effects on all 25 HRV parameters (all *p* < 0.001), with 64% demonstrating exceptionally large effect sizes (*η*^2^p ≥ 0.50). Parasympathetic markers showed the largest responses: pNN50 declined 52% (*η*^2^p = 0.702), HF power declined 51% (*η*^2^p = 0.654), and RMSSD declined 32% (*η*^2^p = 0.420) from low to high density—while sympathetically influenced indices showed substantially smaller effects, confirming parasympathetic withdrawal as the predominant autonomic mechanism. These suppression magnitudes approach levels associated with adverse cardiovascular outcomes. NASA-TLX scores increased 74% across conditions (*p* < 0.001) and correlated with HRV vagal indices (*r* = −0.69 to −0.76), providing corroborating, though not definitive, evidence that crowding engages both subjective and autonomic stress pathways (see Discussion for caveats regarding this association). Temporal analysis confirmed progressive autonomic suppression without habituation across 10-min windows. RoBoSS-SVM achieved 92.2% accuracy for three-class discrimination and 97.2% accuracy (AUC = 0.993) for binary classification, with parasympathetic time-domain features comprising 64% of the optimal feature set. Classroom spatial density produces dose-dependent, mechanism-specific cardiac autonomic dysregulation dominated by vagal withdrawal—consistent with polyvagal theory’s prediction for social-environmental stressors. The excellent classification performance supports feasibility of real-time wearable monitoring for evidence-based classroom capacity management.

**Supplementary Information:**

The online version contains supplementary material available at https://doi.org/10.1186/s40101-026-00451-9.

## Introduction

Student well-being in educational environments has emerged as a critical priority, with international organizations recognizing that optimal learning outcomes depend fundamentally on the physical, psychological, and social conditions within which education occurs [1, 2]. The physical classroom environment encompasses multiple interacting parameters—thermal comfort, indoor air quality, lighting, acoustics, and spatial density—each exerting measurable impacts on student physiological stress, cognitive performance, and learning behaviors [3]. Among these, spatial density—defined as the number of occupants per unit floor area—represents a ubiquitous yet paradoxically understudied environmental stressor [4, 5]. Classroom overcrowding has reached crisis proportions globally, with the United Nations Educational, Scientific and Cultural Organization (UNESCO) estimating that over 300 million students attend schools where density substantially exceeds recommended standards [1, 6]. Recent large-scale evidence confirms that lower-density classrooms significantly enhance school preparedness across cognitive, social-emotional, and physical domains (effect sizes *d* = 0.15–0.32) [7]. Overcrowded classrooms have been shown to create excessive noise levels, reduce personalized instruction opportunities, and increase disciplinary problems [8]. Research consistently demonstrates that overcrowding diminishes both the quantity and quality of teaching and learning, with students in overcrowded settings reporting decreased motivation and feelings of being overlooked [9, 10]. Beyond academic outcomes, overcrowding contributes to teacher burnout, with educators reporting overwhelming workloads and inability to meet diverse student needs [11, 12]. Overcrowding beyond the classroom, including in the home, has likewise been linked to poorer child health, environment, and schooling outcomes, underscoring that crowding-related strain is not confined to purpose-built educational settings [13]. A systematic review and meta-analysis further confirmed that flexible, adequately spaced learning environments significantly enhance student attention and academic achievement compared with high-density constrained settings [14].

The autonomic nervous system (ANS), comprising sympathetic and parasympathetic branches, serves as the primary biological interface between environmental demands and organismal responses [15]. Healthy autonomic function exhibits dynamic flexibility that chronic or acute stress reduces, manifesting as diminished heart rate variability (HRV)—the beat-to-beat fluctuation in cardiac inter-beat intervals [16, 17]. HRV provides a noninvasive, continuous, and objective biomarker of autonomic function, with standardized guidelines established for its measurement and interpretation [18, 19]. Meta-analytic evidence linking reduced RMSSD to 56% heightened all-cause mortality risk (HR = 1.56, 95% CI: 1.32–1.85) underscores the clinical significance of HRV suppression [20], while short-term 5-min recordings have been validated as sufficiently reliable for research and clinical applications [21]. In educational contexts, HRV has demonstrated sensitivity to academic stressors, with examination-related stress associated with significant reductions in SDNN, total power, and HF power alongside increased sympathetic dominance [22]. Concurrently, machine learning has substantially elevated the practical utility of HRV-based stress detection, with state-of-the-art reviews reporting 70–95% classification accuracy across diverse paradigms [23–25]. The RoBoSS (Robust, Bounded, Sparse, Smooth) loss function has demonstrated superior performance specifically on noisy, high-dimensional physiological data—precisely the characteristics of real-world HRV signals [26].

Despite this evidence base, five critical gaps limit current understanding of classroom density as a physiological stressor. First, educational crowding research has relied exclusively on subjective self-reports and academic performance metrics without concurrent objective cardiac autonomic assessment [11, 27, 28]. Second, existing physiological crowding investigations employ observational designs that preclude causal inference, as confounding by socioeconomic status, facility quality, and population heterogeneity cannot be controlled [29]. Third, studies employ binary crowded-versus-uncrowded comparisons rather than systematic multi-level density manipulation, preventing the dose-response characterization essential for establishing evidence-based capacity standards [6]. Fourth, crowding research remains largely atheoretical, failing to specify the autonomic mechanism—sympathetic activation versus parasympathetic withdrawal—within established frameworks such as polyvagal theory or the neurovisceral integration model [30]. Fifth, no density-specific machine learning classification model enabling automated real-time monitoring has been developed or validated, preventing translation of research findings into institutional practice [31].

The present investigation is theoretically grounded in two complementary neuroscientific frameworks. The neurovisceral integration model posits that central autonomic networks—encompassing the prefrontal cortex, anterior cingulate cortex, and amygdala—regulate cardiac vagal tone through descending inhibitory pathways, predicting that environmental threat perception produces dose-response vagal withdrawal manifesting as reductions in HF power, RMSSD, pNN50, and CVI [15]. Polyvagal theory further specifies that crowded environments generate simultaneous threat cues—violated interpersonal space, elevated noise, and reduced perceived control— that compromise the ventral vagal complex, producing predominantly parasympathetic suppression rather than sympathetic co-activation [16, 32]. This distinction between social-environmental stressors and acute life-threatening stimuli is well-established within the polyvagal framework [33]. Empirical evidence confirming that autonomic state shifts reflect defensive responses to perceived environmental threat provides direct support for this theoretical account [34].

The present investigation addressed these five gaps through a within-subjects experiment manipulating classroom density at three calibrated levels (30, 60, and 100 students/150 m^2^) while continuously controlling environmental parameters—temperature, humidity, noise, and illuminance—to enable unconfounded causal inference. The experimental protocol was informed by established recommendations for continuous indoor environmental monitoring during teaching activities [35]. Twenty-five HRV parameters spanning time-domain, frequency-domain, and nonlinear domains were extracted following standardized measurement and reporting guidelines [18, 19], complemented by validated NASA Task Load Index subjective workload assessment [36, 37]. Temporal segmentation into three 10-min windows per session enabled characterization of both dose-response relationships and within-session temporal dynamics. RoBoSS-SVM with Recursive Feature Elimination provided density-specific classification for automated monitoring feasibility assessment [26].

This investigation pursued five aims: to quantify the dose-dependent effects of the three density conditions on all 25 HRV parameters; to identify the primary autonomic mechanism—sympathetic activation versus parasympathetic withdrawal—through effect-size comparisons across ANS-specific indices; to characterize temporal autonomic dynamics across the three 10-min windows within each session; to validate crowding as a psychophysiological stressor through convergent HRV-NASA-TLX analysis; and to develop and validate a density-specific RoBoSS-SVM classifier for real-time monitoring feasibility. Based on polyvagal theory and the neurovisceral integration model, four directional hypotheses were formulated. It was hypothesized that parasympathetic withdrawal would dominate over sympathetic activation, with vagal-specific indices showing larger effect sizes than sympathetically influenced parameters [16, 32]. It was further hypothesized that HRV parameters would follow continuous linear dose-response gradients across all three density levels—including significant differences between low and medium conditions—indicating physiological strain even at moderate crowding [4, 6]. Regarding temporal dynamics, autonomic suppression was expected to intensify progressively across 10-min windows without habituation, consistent with depletion of self-regulatory resources under sustained environmental stress [38]. Finally, RoBoSS-SVM was hypothesized to exceed 85% accuracy for three-class and 95% for binary discrimination, with parasympathetic time-domain features dominating the optimal feature subset [24, 26].

To our knowledge, this is the first controlled experimental study to employ continuous multi-domain HRV monitoring specifically for classroom density characterization [27], simultaneously specifying the autonomic mechanism within established theoretical frameworks [15, 16], characterizing within-session temporal dynamics, and establishing machine learning classification feasibility for automated monitoring [24, 26]—advancing both the theoretical understanding of environmental stress physiology and the practical development of evidence-based educational capacity standards and student physiological monitoring systems [31].

## Materials and methods

### Study design and participants

This cross-sectional experimental study was conducted at Suez University, Egypt, between Sep and Dec 2025, employing a within-subjects repeated-measures design to maximize statistical power while minimizing interindividual variability [39]. A within-subjects design was selected because it controls for individual differences in baseline autonomic function, substantially increasing sensitivity to density-induced effects.

Sample size was determined using G*Power 3.1 [40, 41] for a one-way within-subjects repeated-measures ANOVA with three measurement levels, *α* = 0.05, power = 0.95, anticipated large effect size (Cohen’s *f* = 0.40) based on comparable psychophysiological crowding studies, and estimated inter-condition correlation *r* = 0.50. The minimum required sample was 28 participants; 30 were recruited (achieved power = 0.97) to provide a 6.7% buffer against potential dropout [42].

Thirty healthy university students (16 males, 14 females; age range 18–25 years, mean 22.3 ± 2.1 years) were recruited through campus advertisements. Inclusion criteria comprised age 18–25 years, full-time student status, no cardiovascular, respiratory, or neurological disease, no medications affecting autonomic function, BMI 18.5–29.9 kg/m^2^, resting heart rate 60–100 bpm, and ability to attend three sessions separated by ≥7 days [43]. Exclusion criteria included psychiatric disorders requiring pharmacological treatment, chronic diseases affecting autonomic function, pregnancy, active smoking, excessive alcohol consumption (>14 drinks/week), caffeine or alcohol intake within 4 h of sessions, strenuous exercise within 24 h, and sleep duration <6 or >9 h preceding sessions [19]. Baseline demographic and physiological characteristics are presented in Table 1. Table S1 presents the complete CONSORT-adapted participant recruitment and retention flow.

**Table 1:** Baseline demographic and physiological characteristics of study participants (*N* = 30)

Parameter	Total sample (*N* = 30)	Males (*n* = 16)	Females (*n* = 14)	*p* value
Age (years)	22.3 ± 2.1 (18–25)	22.4 ± 2.2	22.2 ± 1.9	0.751
Gender distribution	–	16 (53.3%)	14 (46.7%)	–
BMI (kg/m^2^)	24.1 ± 3.2	24.3 ± 3.1	23.8 ± 3.3	0.658
Resting HR (bpm)	68.5 ± 8.2	67.2 ± 8.0	70.1 ± 7.8	0.298
Systolic BP (mmHg)	118 ± 9	118.1 ± 9.2	116.3 ± 8.7	0.567
Diastolic BP (mmHg)	74 ± 7	75.8 ± 7.1	74.2 ± 6.9	0.514
Physical activity (h/week)	–	4.1 ± 2.2	3.5 ± 2.0	0.412

### Experimental protocol

**Fig. 1:**
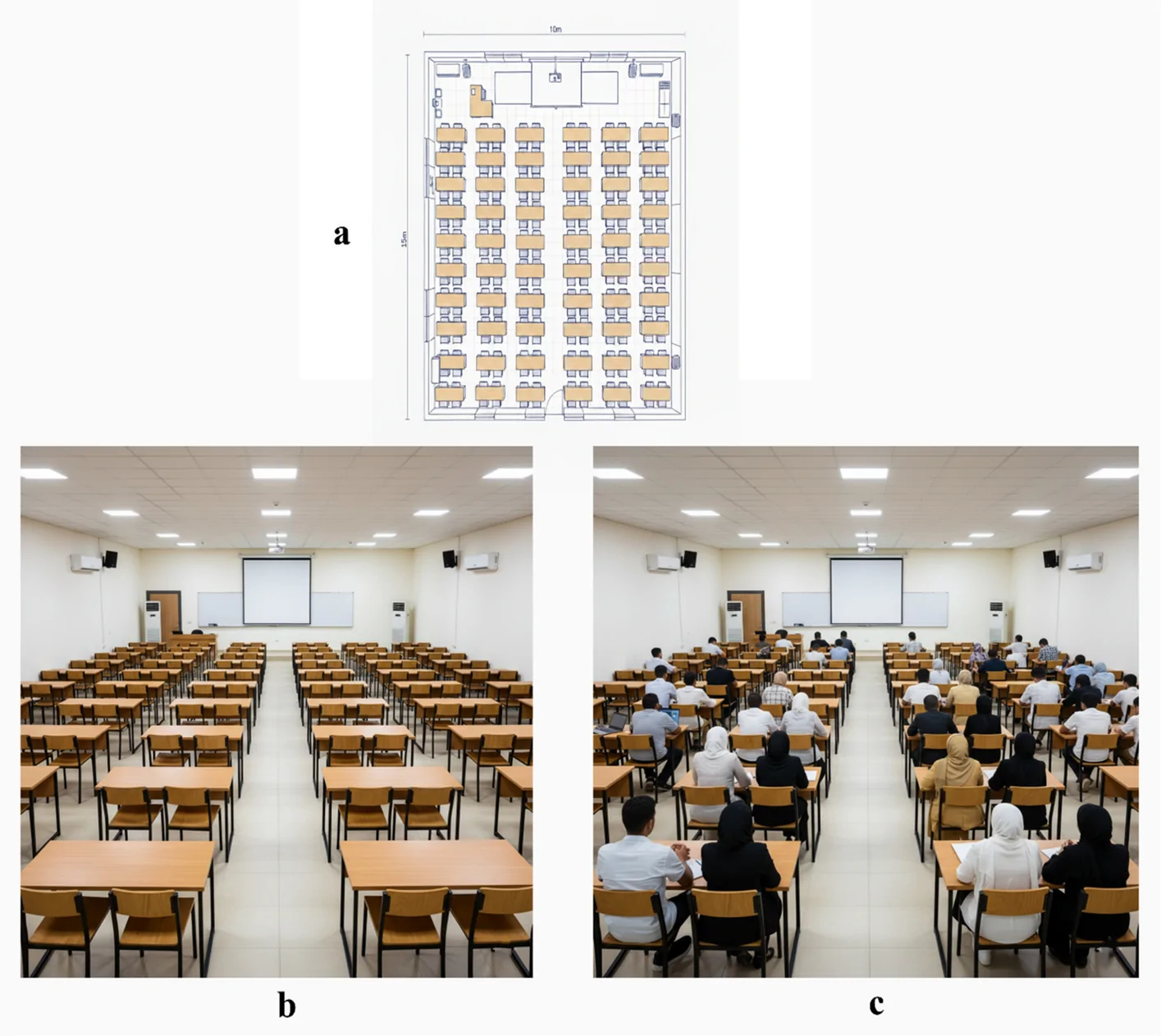
Classroom environment and density manipulation: **a** floor plan showing standardized classroom dimensions (150 m^2^); **b** empty condition; **c** high-density condition

Each participant completed three experimental sessions corresponding to low density (30 students/150 m^2^), medium density (60 students/150 m^2^), and high density (100 students/150 m^2^). These three levels were selected to represent comfortable, moderately crowded, and severely overcrowded conditions based on international educational standards and prior research [4, 35]. All sessions were conducted in a standardized classroom (150 m^2^) with continuously monitored environmental conditions (Fig. 1). Density manipulation was achieved by adjusting the number of confederate participants while maintaining constant classroom dimensions and furniture arrangement.

Sessions were scheduled between 09:00 and 12:00 to control for circadian variations in HRV [44]. Session order was counterbalanced across participants using a Latin square design, and inter-session intervals of ≥7 days minimized carryover effects.

**Fig. 2:**
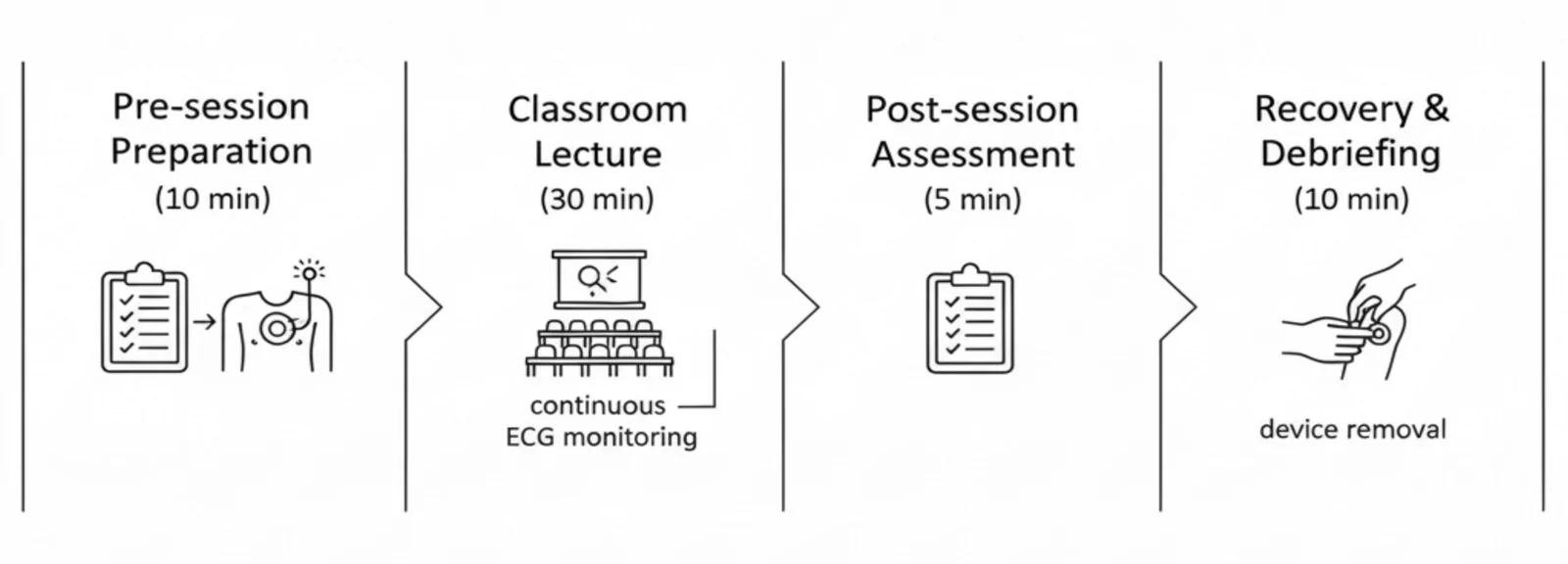
Experimental protocol timeline showing the four sequential phases of each experimental session

Each session followed a standardized four-phase protocol (Fig. 2): phase 1 (pre-session preparation, 10 min): participants completed screening questionnaires and baseline NASA-TLX workload assessment [37], followed by ECG electrode placement with real-time signal quality verification.

Phase 2 (classroom lecture, 30 min): continuous ECG monitoring during a standardized lecture on Introduction to Descriptive Statistics, delivered by the same instructor across all sessions. Phase 3 (post-session assessment, 5 min): re-administration of NASA-TLX to assess experienced workload. Phase 4 (recovery and debriefing, 10 min): cardiovascular recovery monitoring, device removal, and participant debriefing [45].

Participants adhered to standardized pre-session behavioral restrictions: 7–9 h of sleep, no caffeine within 4 h, no alcohol within 24 h, no strenuous exercise within 24 h, and a light breakfast ≥1 h before sessions [46]. Environmental parameters—temperature, relative humidity, noise level, and illuminance—were continuously monitored using calibrated sensors throughout all sessions [35]. Repeated-measures ANOVA confirmed no significant differences across density conditions for any environmental parameter (all *p* > 0.05), confirming that observed HRV effects were attributable specifically to interpersonal density (Table 2).

**Table 2:** Environmental parameters across classroom density conditions

Parameter	Low density (30 students)	Medium density (60 students)	High density (100 students)	*p* value
Temperature (°C)	23.2 ± 0.8	23.5 ± 0.9	23.8 ± 1.0	0.159
Relative humidity (%)	46.5 ± 3.5	47.2 ± 3.8	48.1 ± 4.2	0.473
Noise level (dB)	44.8 ± 3.2	46.3 ± 3.7	47.9 ± 4.1	0.108
Illuminance (lux)	448 ± 22	451 ± 26	449 ± 24	0.955

### ECG data acquisition

**Fig. 3:**
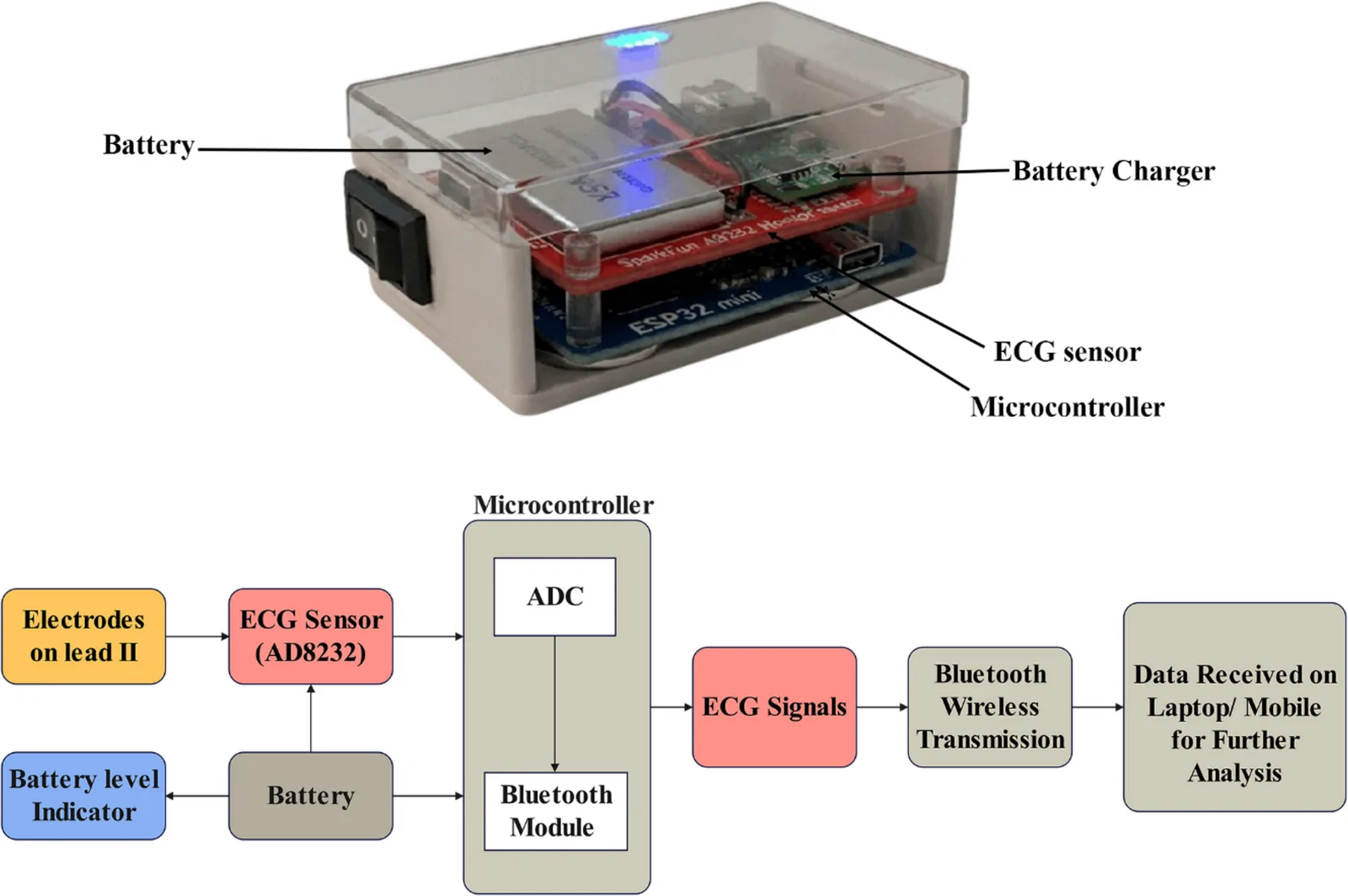
Custom-designed wearable ECG monitoring device; 3D view showing compact patch-type housing; and internal architecture block diagram

ECG signals were acquired using a custom-designed wearable monitoring device integrating an AD8232 single-lead heart rate monitor analog front-end (Analog Devices, Norwood, MA, USA), configured with 100× gain, integrated high-pass filtering (0.5 Hz cutoff), low-pass filtering (40 Hz cutoff), and right-leg drive circuit for common-mode interference rejection [47]. The analog output was digitized using a 12-bit analog-to-digital converter (ADC) at 100 Hz sampling frequency within an ESP32 microcontroller (Espressif Systems, Shanghai, China), with real-time data transmitted via Bluetooth Low Energy at 1.2 kbps [48]. The compact patch-type device (55 × 35 × 20 mm, 45 g) was powered by a rechargeable lithium-ion battery (3.7 V, 1000 mAh) providing >3 h of continuous operation. Figure 3 shows the custom-designed wearable ECG device architecture and electrode configuration.

Two dry stainless steel electrodes (10 mm diameter, 316L surgical-grade) were positioned in modified lead II configuration: negative electrode on the right upper chest below the clavicle, positive electrode on the left lower chest near the cardiac apex—a configuration providing excellent R-wave amplitude due to favorable cardiac axis alignment. Prior to each recording, electrode sites were cleansed with 70% isopropyl alcohol and electrode-skin impedance was verified to be <10 kΩ per electrode using a bioimpedance analyzer.

**Fig. 4:**
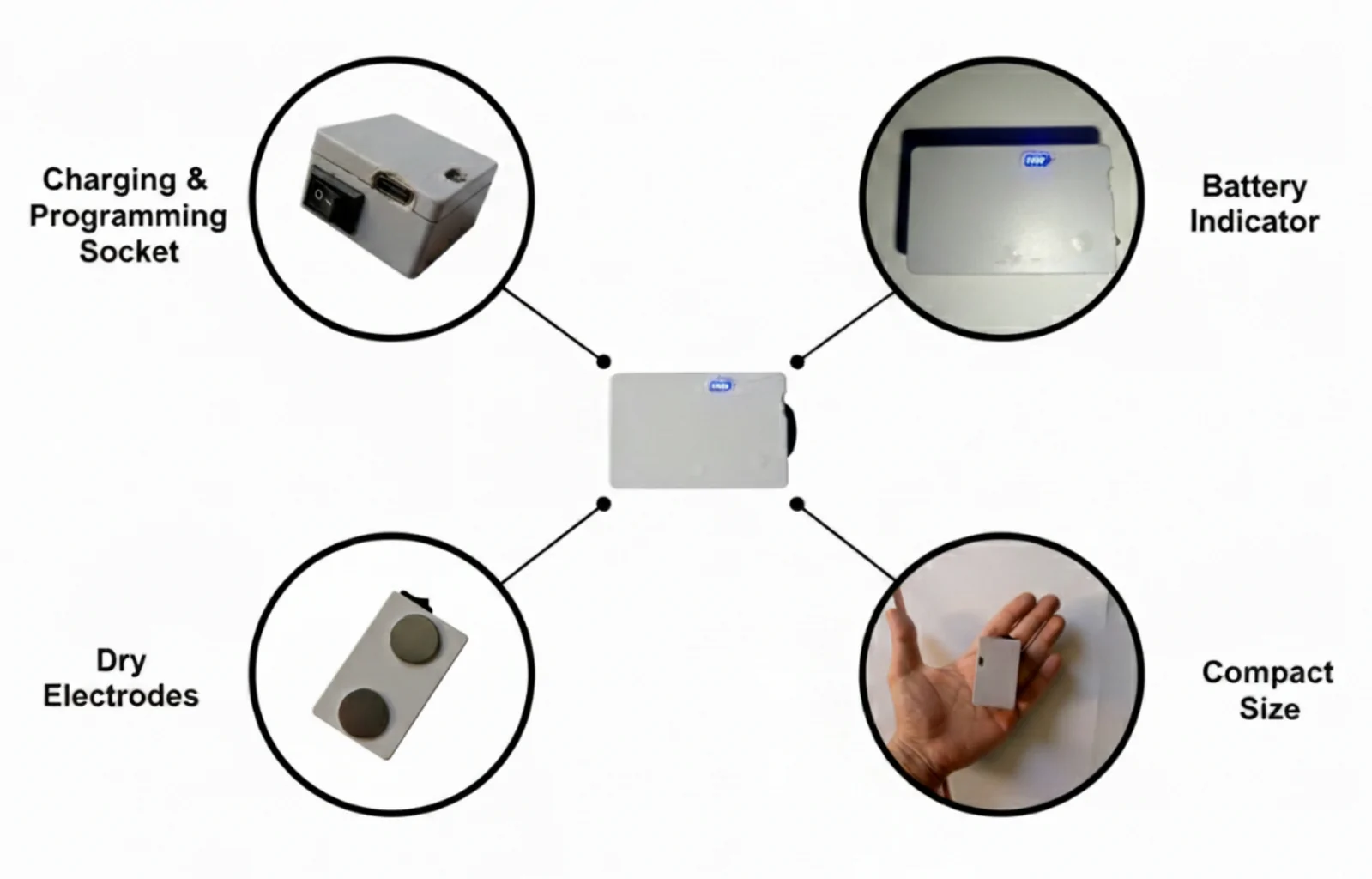
Key features of the wearable ECG device

**Fig. 5:**
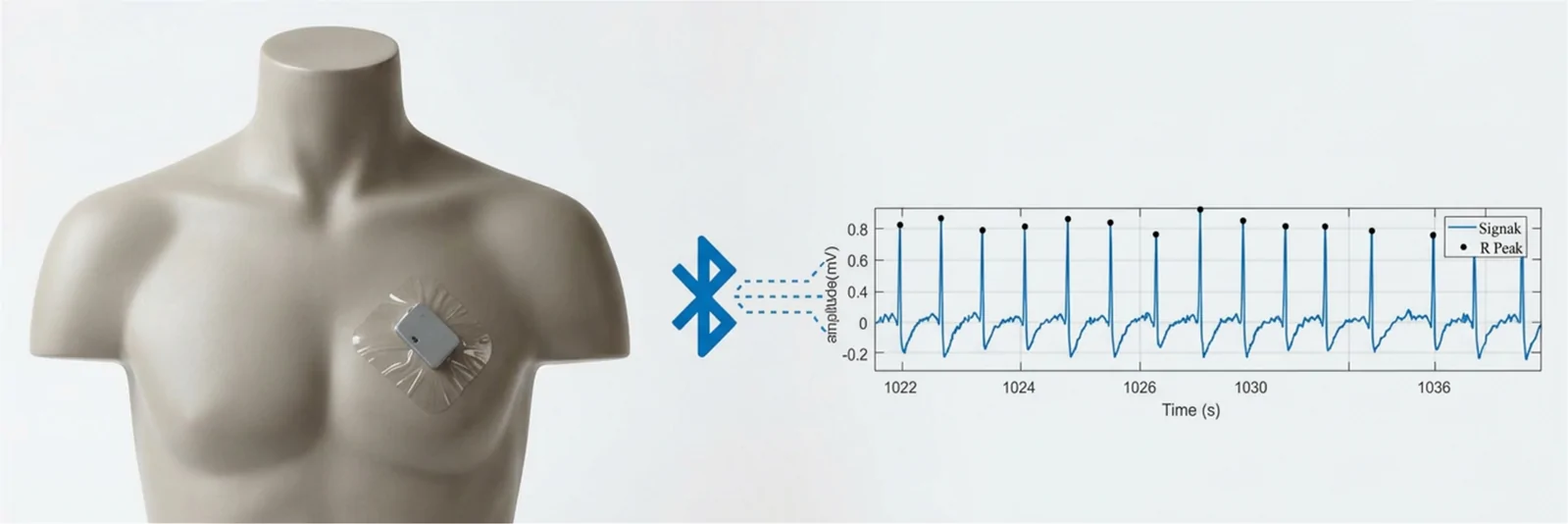
In situ ECG monitoring setup: wearable device placement on student’s chest in modified lead II configuration; representative real-time ECG signal showing clear R-wave amplitude; and Bluetooth wireless transmission to data acquisition system

Device validation was conducted in ten volunteers under simultaneous recording with a medical-grade electrocardiograph (iE 300 Digital 3-Channel ECG). Comparative analysis demonstrated excellent RR interval agreement (Bland–Altman bias +1.2 ms, 95% limits of agreement −8.4 to +10.8 ms). During the main study, mean signal-to-noise ratio was 38.7 ± 4.3 dB (range: 28.2–49.1 dB), substantially exceeding the 20 dB minimum recommended for automated R-peak detection, with a mean artifact rate of 3.2 ± 1.8%. The key structural features of the wearable device are illustrated in Fig. 4, while the practical monitoring setup, electrode placement, and real-time signal acquisition are demonstrated in Fig. 5.

### Signal preprocessing and RR interval extraction

Raw ECG signals were preprocessed offline using MATLAB R2023b. A zero-phase finite impulse response (FIR) bandpass filter (0.5–40 Hz) was applied using the Parks-McClellan optimal equiripple design to remove baseline wander and attenuate noise while preserving QRS morphology. A supplementary 50 Hz notch filter (*Q* = 30) eliminated residual power-line interference.

R-peak detection employed the Pan–Tompkins algorithm, incorporating differentiation, squaring, moving-window integration, adaptive dual thresholding, and physiological constraint verification [49]. All automatically detected R-peaks underwent independent visual inspection by two trained personnel blinded to experimental conditions; interrater agreement exceeded 98.5% (Cohen’s *κ* = 0.97). The RR interval time series was constructed by computing successive differences between adjacent R-peak locations, expressed in milliseconds with 10 ms resolution.

**Fig. 6:**
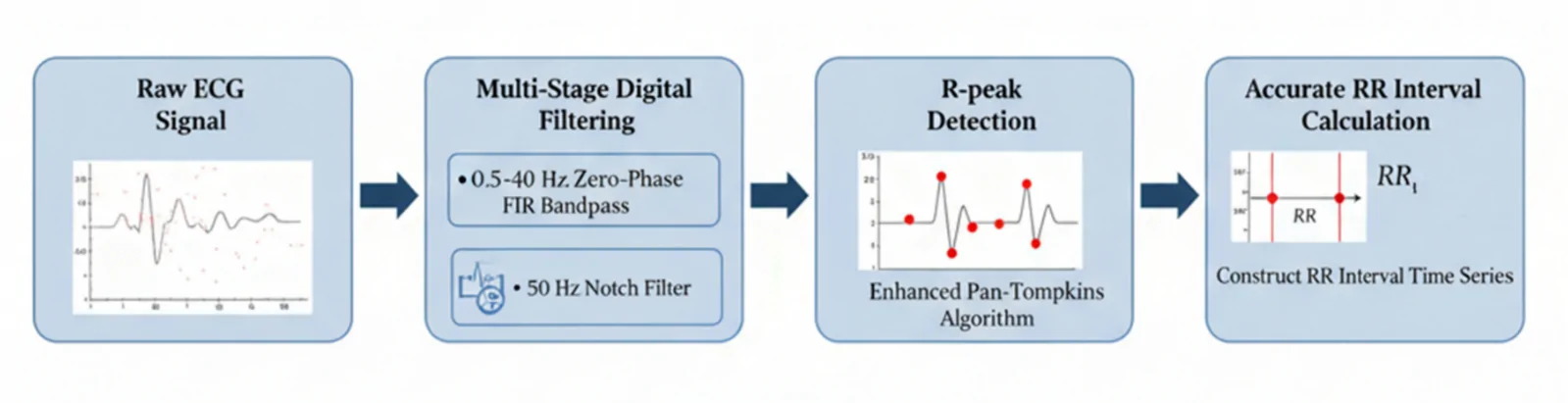
Signal preprocessing pipeline from raw ECG acquisition to RR interval extraction

Artifactual RR intervals were identified and removed using three complementary criteria: physiological range filtering (300–2000 ms), sudden change detection (>20% difference from the preceding interval), and statistical outlier detection (>3 SD from a moving median). Segments containing >5% artifacts were entirely excluded from analysis; no segments met this exclusion criterion in the present dataset. Figure 6 presents the complete signal preprocessing pipeline from raw ECG to RR interval extraction.

### Heart rate variability parameter extraction

**Fig. 7:**
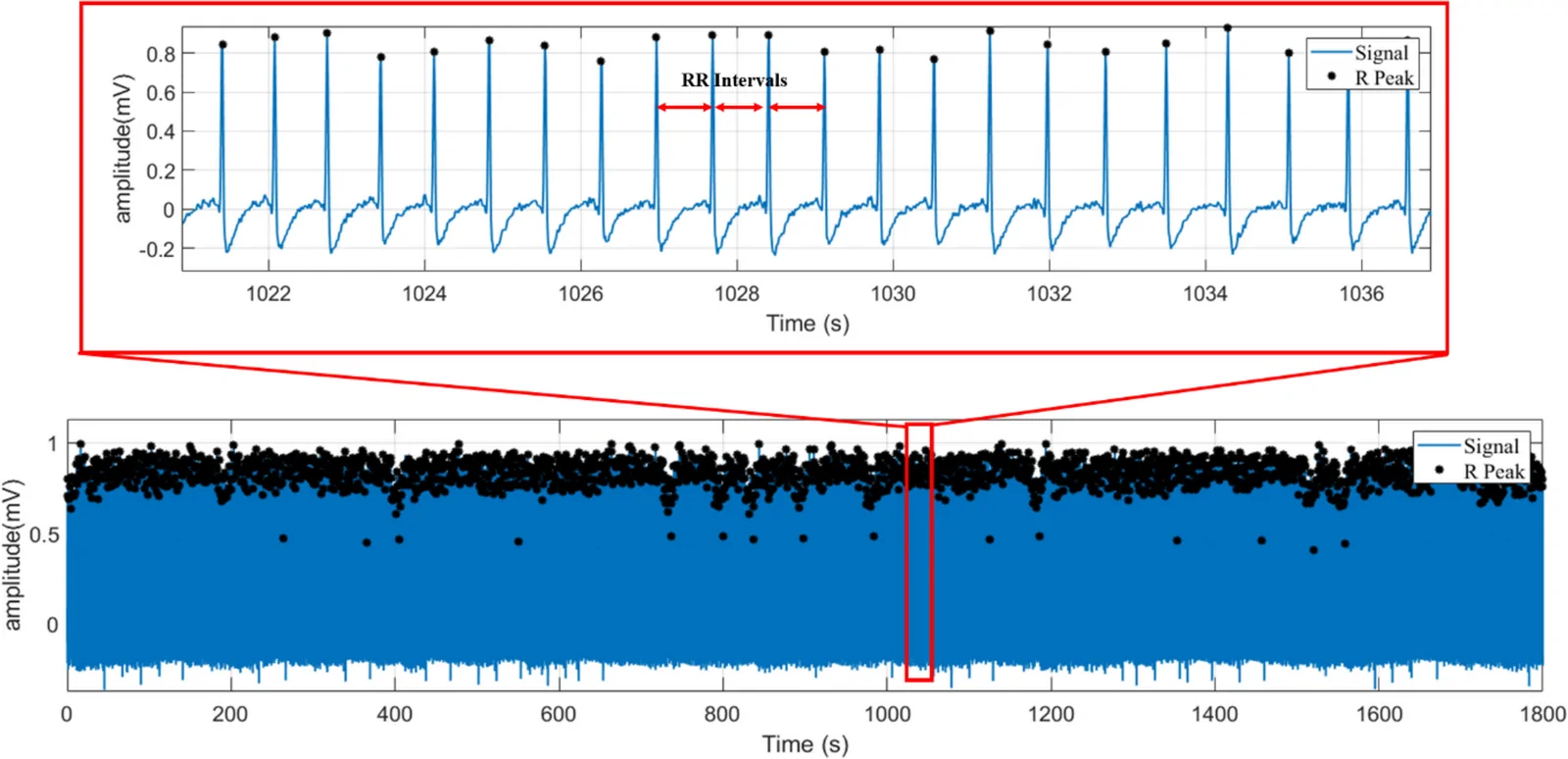
Representative 30-min continuous ECG recording

HRV analysis was performed on three consecutive non-overlapping 10-min segments extracted from each 30-min lecture session (Fig. 7). This segmentation strategy satisfies the 10-min minimum duration recommended by the Task Force for reliable short-term HRV assessment [18], increases statistical power by yielding 270 analysis segments, and enables temporal dynamics characterization. All parameters were computed using validated algorithms from the PhysioNet Cardiovascular Signal Toolbox [50].

Twenty-five HRV parameters were calculated across three analytical domains to provide comprehensive cardiac autonomic characterization. Time-domain parameters (*n* = 8) were computed using standard Task Force formulas: mean RR interval (ms; the interval between successive R waves of the electrocardiographic QRS complex), mean HR (bpm; heart rate), SDNN (ms; standard deviation of NN [normal-to-normal] intervals) representing total HRV magnitude, RMSSD (ms; root mean square of successive differences between adjacent NN intervals) indicating cardiac parasympathetic activity, NN50 (count; number of successive NN interval pairs differing by more than 50 ms) and pNN50 (%; NN50 expressed as a percentage of total NN intervals) highly specific for parasympathetic function, SDANN (ms; standard deviation of the averages of NN intervals across short segments) reflecting longer-term variability, and SDNN Index (ms; the mean of the short-segment SDNN values) [18]. Frequency-domain parameters (*n* = 7) were extracted using autoregressive modeling (Burg method, order = 16): VLF (very low frequency power, from 0.003 to 0.04 Hz), LF (form low frequency power, 0.04 to 0.15 Hz), HF (from high frequency power, 0.15 to 0.40 Hz), total power (ms^2^), LF/HF ratio, and LF and HF normalized units (n.u.) [51]. Nonlinear parameters (*n* = 10) were calculated: Poincaré plot parameters (SD1, reflecting short-term/instantaneous beat-to-beat variability; SD2, reflecting long-term variability; and the SD1/SD2 ratio), sample entropy (SampEn), approximate entropy (ApEn), correlation dimension (D2), detrended fluctuation analysis (DFA α1 and α2), cardiac sympathetic index (CSI), and cardiac vagal index (CVI) [52]. Table S2 provides comprehensive definitions, formulas, and physiological interpretations for all 25 parameters; the List of Abbreviations provides a complete reference for all abbreviations used in this manuscript.

For readers across disciplines, we clarify the autonomic polarity of each parameter here. An increase in the parasympathetic (vagal) markers—RMSSD, pNN50, NN50, SDNN, SDANN, SDNN Index, HF power, HF n.u., SD1, SD2, CVI, SampEn, and ApEn—indicates greater parasympathetic activity, whereas a decrease indicates parasympathetic withdrawal. Conversely, an increase in LF power, LF n.u., the LF/HF ratio, DFA α1, and CSI is generally interpreted as reflecting relatively greater sympathetic or sympathovagal dominance, whereas a decrease indicates reduced sympathetic influence. Applying this convention, the pattern observed in this study—a decline in parasympathetic markers alongside comparatively small changes in sympathetic markers—indicates that parasympathetic withdrawal, rather than sympathetic activation, was the dominant autonomic response (Effect size analysis and autonomic mechanism specification section).

### Feature selection

Feature selection was performed to identify the optimal HRV parameter subset maximizing classification accuracy while minimizing model complexity. Recursive Feature Elimination (RFE) was employed due to its wrapper-based approach that directly optimizes classification performance, avoiding the limitations of filter methods that assess feature relevance independently of the classifier [53]. RFE was embedded within a stratified group-based fivefold cross-validation framework. All three temporal segments from the same participant were constrained to remain within the same fold, preventing data leakage from temporal dependencies and ensuring genuine participant-level generalization assessment [54]. At each iteration, the feature with the lowest RFE-assigned importance score was eliminated, and classification accuracy was recorded as a function of feature subset size to identify the optimal number of features.

### RoBoSS-SVM classification

Support Vector Machine with the Robust, Bounded, Sparse, and Smooth (RoBoSS) loss function was employed as the primary classification algorithm. Standard SVM hinge loss lacks robustness to outliers, which can disproportionately influence decision boundaries in noisy physiological data. The RoBoSS loss function addresses these limitations through a mathematical reformulation ensuring four simultaneous properties: robustness—bounding the loss to prevent extreme penalties from artifactual observations; sparsity—zero loss for correctly classified samples enabling automatic feature weighting; smoothness—differentiability everywhere ensuring efficient gradient-based optimization; and boundedness—upper-bounding the loss by 1/*a*, preventing unbounded influence of noise-contaminated samples [26].

The RoBoSS loss function is formally defined for functional margin *z* = *y*_*i*_ *f*(*x*_*i*_) as [26]:

1 $${L}_{rbss}\left(u\right)=\left\{\begin{array}{c}\lambda \left(1-\left(au+1\right){e}^{-au}\right), u>0,\\ 0\ \quad\quad\quad\quad\quad\quad\quad\quad ,u\le 0, \end{array}\right.$$

where *a* > 0 is a shape parameter controlling loss curvature and *λ* > 0 is a bounding parameter controlling the maximum loss value for misclassified samples. The RBF kernel was selected for its universal approximation capability and empirically superior performance across diverse HRV classification benchmarks [26]. Table S3 provides a direct mathematical comparison of RoBoSS-SVM against standard SVM across the four key properties. Comprehensive evaluations across 88 benchmark datasets confirmed that RoBoSS-SVM achieves significant accuracy improvements over standard SVM, with the largest gains on noisy, high-dimensional data [26].

Two classification scenarios were investigated. Scenario 1—three-class discrimination (low vs. medium vs. high): all 270 segments (90 per level) using one-versus-rest multi-class strategy. Scenario 2—binary classification (low vs. high): 180 segments (90 per extreme density level), excluding medium density data, to assess maximal discriminative capacity. Hyperparameter optimization via exhaustive grid search identified optimal regularization *C* ∈ {0.1, 1, 10, 50, 100} and RBF coefficient *γ* ∈ {0.001, 0.01, 0.1, 0.5, 1.0} [26].

### Statistical analysis

All statistical analyses were conducted using Python 3.13 with a family-wise significance threshold of *α* = 0.05. Effect sizes were reported alongside *p* values following contemporary recommendations for transparent statistical reporting [42, 55].

Normality of data distributions was assessed prior to parametric testing using the Shapiro–Wilk test, which is the most powerful omnibus normality test for sample sizes of *N* ≤ 50 [56]. Q-Q plots and skewness/kurtosis statistics (acceptable range: |skewness| < 2.0, |kurtosis| < 7.0) were examined as supplementary normality indicators [55]. All 25 HRV parameters satisfied normality assumptions at all three density conditions (Shapiro–Wilk *W* = 0.934–0.981, all *p* > 0.05), confirming the appropriateness of parametric repeated-measures ANOVA. Table S4 presents normality test results for all parameters.

Mauchly’s test of sphericity was conducted for each repeated-measures ANOVA to evaluate equal variances among all pairwise difference scores [55]. Where Mauchly’s test indicated significant violation (*p* < 0.05), degrees of freedom were corrected using the Greenhouse–Geisser (GG) epsilon (*ε*) correction to prevent type I error inflation [57]. Four parameters showed sphericity violations (pNN50, HF power, SampEn, CSI; all Mauchly’s *p* < 0.05) and received GG correction; all remained significant at *p* < 0.001 after correction, confirming robustness of findings. Table S5 presents sphericity test results for affected parameters.

One-way repeated-measures ANOVA evaluated density effects (low, medium, high) on each of the 25 HRV parameters, controlling for individual differences to maximize statistical power. Effect sizes were quantified using partial eta squared (*η*^2^p), computed as:

η^2^p = SS_effect / (SS_effect + SS_error)

where SS_effect is the sum of squares for the density main effect and SS_error is the within-subjects error sum of squares. Unlike *η*^2^, *η*^2^p is the preferred effect size for within-subjects designs as it expresses each effect’s variance relative only to its own error term, providing stable cross-study comparisons [40, 55]. Interpretation followed Cohen’s (1988) benchmarks as operationalized for *η*^2^p: small (≥0.01), medium (≥0.06), large (≥0.14) [42]. Table S6 presents the effect size interpretation framework and the distribution of parameters across categories in this study.

When ANOVA revealed significant main effects (*p* < 0.05), post hoc pairwise comparisons were conducted using Bonferroni correction. For three pairwise comparisons (low–medium, low–high, medium–high), the adjusted significance threshold was:

α_adjusted = α_familywise/*k* = 0.05/3 = 0.0167

where *k* = 3 is the number of pairwise comparisons and α_familywise = 0.05 is the desired family-wise error rate [55]. All reported post hoc significance designations (*p < 0.05, **p < 0.01, ***p < 0.001) reflect comparisons against this adjusted threshold. Pearson correlations assessed linear associations between NASA-TLX and objective HRV parameters across all observations (*n* = 90). Classifier performance was evaluated using stratified group-based fivefold cross-validation with participant-level holdout, reporting accuracy, sensitivity, specificity, precision, F1-score, and ROC-AUC for each classification scenario.

## Results

### Density-driven cardiac autonomic dysregulation

All 25 HRV parameters demonstrated statistically significant main effects of classroom spatial density (one-way repeated-measures ANOVA; all *F*(2,58) values ranging from 8.2 to 68.2; all *p* < 0.001; Table 3). Critically, 64% of parameters (16/25) demonstrated exceptionally large effect sizes (*η*^2^p ≥ 0.50), substantially exceeding Cohen’s benchmark for large effects (*η*^2^p ≥ 0.14) and the typical range observed in psychophysiological intervention research. The remaining 36% of parameters demonstrated large-to-very large effects (*η*^2^p = 0.148–0.498), confirming that no parameter exhibited only small or moderate responses to density manipulation [42].

**Table 3:** Descriptive statistics and repeated-measures ANOVA results for all 25 HRV parameters across three classroom density conditions (*N* = 30)

HRV parameter	Unit	Low density M ± SD	Medium density M ± SD	High density M ± SD	*F*(2,58)	*p*	*η*^2^p	% change (L → H)
Time-domain parameters								
Mean RR	ms	880.2 ± 85.3	831.8 ± 78.2	768.4 ± 91.6	38.2	<0.001	0.569	−12.7%
Mean HR	bpm	68.5 ± 8.2	72.1 ± 7.8	78.3 ± 9.1	42.1	<0.001	0.592	+14.3%
SDNN	ms	58.3 ± 12.4	45.2 ± 10.8	36.8 ± 9.2	52.8	<0.001	0.583	−36.9%
RMSSD	ms	42.5 ± 11.2	34.8 ± 9.6	28.9 ± 8.4	31.4	<0.001	0.420	−32.0%
pNN50	%	28.4 ± 8.5	18.6 ± 7.2	13.6 ± 5.8	68.2	<0.001	0.702	−52.1%
NN50	Count	142.3 ± 44.8	93.2 ± 37.6	67.8 ± 30.5	58.3	<0.001	0.668	−52.4%
SDANN	ms	52.1 ± 11.8	41.3 ± 10.2	33.8 ± 8.7	44.6	<0.001	0.607	−35.1%
SDNN Index	ms	38.6 ± 9.3	31.2 ± 8.1	25.4 ± 7.2	35.8	<0.001	0.498	−34.2%
Frequency-domain parameters								
VLF power	ms^2^	892.3 ± 244.8	712.4 ± 198.2	528.6 ± 175.8	28.4	<0.001	0.396	−40.8%
LF power	ms^2^	728.4 ± 198.2	631.2 ± 172.4	518.3 ± 147.6	24.6	<0.001	0.362	−28.8%
HF power	ms^2^	482.3 ± 141.8	312.4 ± 107.6	238.2 ± 91.4	58.7	<0.001	0.654	−50.6%
Total power	ms^2^	2102.4 ± 512.3	1655.2 ± 444.8	1284.8 ± 384.6	36.2	<0.001	0.512	−38.9%
LF/HF ratio	–	1.5 ± 0.5	2.0 ± 0.6	2.2 ± 0.6	18.3	<0.001	0.285	+43.4%
LF n.u.	n.u.	48.2 ± 10.4	55.8 ± 11.2	59.2 ± 12.1	22.4	<0.001	0.327	+22.8%
HF n.u.	n.u.	51.8 ± 10.2	44.2 ± 9.8	40.8 ± 11.3	20.1	<0.001	0.302	−21.2%
Nonlinear parameters								
SD1	ms	30.1 ± 8.4	24.6 ± 7.2	20.4 ± 6.8	31.6	<0.001	0.422	−32.2%
SD2	ms	72.4 ± 18.2	56.8 ± 15.4	45.8 ± 13.2	42.8	<0.001	0.596	−36.7%
SD1/SD2	–	0.4 ± 0.1	0.4 ± 0.1	0.4 ± 0.1	8.2	0.001	0.148	+7.2%
SampEn	a.u.	1.8 ± 0.4	1.5 ± 0.3	1.3 ± 0.3	38.4	<0.001	0.570	−29.7%
ApEn	a.u.	1.3 ± 0.2	1.1 ± 0.2	0.9 ± 0.2	32.6	<0.001	0.440	−28.1%
D2	a.u.	2.8 ± 0.5	2.4 ± 0.5	2.1 ± 0.4	28.8	<0.001	0.401	−26.8%
DFA α1	–	1.1 ± 0.2	1.3 ± 0.2	1.4 ± 0.2	26.4	<0.001	0.378	+26.8%
DFA α2	–	1.0 ± 0.1	1.1 ± 0.2	1.2 ± 0.2	22.8	<0.001	0.332	+20.4%
CSI	a.u.	2.4 ± 0.6	2.8 ± 0.7	3.1 ± 0.8	16.2	<0.001	0.248	+29.5%
CVI	a.u.	3.3 ± 0.4	3.0 ± 0.4	2.7 ± 0.3	48.4	<0.001	0.626	−17.1%

*Abbreviations*: *SDNN* standard deviation of NN intervals, *RMSSD* root mean square of successive differences, *NN50/pNN50* count/percentage of successive NN interval differences > 50 ms, *SDANN* standard deviation of the averages of NN intervals, *VLF/LF/HF* very-low-/low-/high-frequency power, *n.u.* normalized units, *SD1/SD2* Poincaré plot short-/long-term variability, *SampEn/ApEn* sample/approximate entropy, *D2* correlation dimension, *DFA* detrended fluctuation analysis, *CSI/CVI* cardiac sympathetic/vagal index, *η*^*2*^*p* partial eta squared

Post hoc Bonferroni analysis revealed significant pairwise differences between all three density levels for 23 of 25 parameters (*p* < 0.0167 corrected threshold), including significant low versus medium comparisons for all vagal-specific indices—confirming that physiologically meaningful autonomic strain occurs at moderate crowding (60 students/150 m^2^), not exclusively at extreme density. Two parameters (SD1/SD2 ratio and DFA α2) showed significant low-vs-high and medium-vs-high differences only (*p* < 0.0167), indicating threshold-like responses at high density. Table 4 summarizes the post hoc pairwise significance pattern across all parameters.

**Table 4:** Post hoc Bonferroni pairwise comparisons—significance summary across all 25 HRV parameters

HRV parameter	Low vs. medium (*p* < 0.0167)	Low vs. high (*p* < 0.0167)	Medium vs. high (*p* < 0.0167)	All pairs significant?
Time-domain (8)				
Mean RR	***	***	***	✓ Yes
Mean HR	***	***	***	✓ Yes
SDNN	***	***	***	✓ Yes
RMSSD	**	***	***	✓ Yes
pNN50	***	***	***	✓ Yes
NN50	***	***	***	✓ Yes
SDANN	***	***	***	✓ Yes
SDNN Index	**	***	***	✓ Yes
Frequency-domain (7)				
VLF power	**	***	***	✓ Yes
LF power	*	***	***	✓ Yes
HF power	***	***	***	✓ Yes
Total power	***	***	***	✓ Yes
LF/HF ratio	**	***	*	✓ Yes
LF n.u.	**	***	**	✓ Yes
HF n.u.	**	***	**	✓ Yes
Nonlinear (10)				
SD1	**	***	***	✓Yes
SD2	***	***	***	✓ Yes
SD1/SD2 ratio	ns	*	*	✗ No (L-M ns)
SampEn	***	***	***	✓ Yes
ApEn	**	***	***	✓ Yes
D2	**	***	**	✓ Yes
DFA α1	**	***	**	✓ Yes
DFA α2	ns	***	**	✗ No (L-M ns)
CSI	*	***	**	✓ Yes
CVI	***	***	***	✓ Yes

Note. *p < 0.05; **p < 0.01; ***p < 0.001 (all against Bonferroni-corrected threshold *α* = 0.0167). *ns*, not significant. ✓ = all three pairwise comparisons significant, including low vs. medium

### Time-domain HRV parameters

**Fig. 8:**
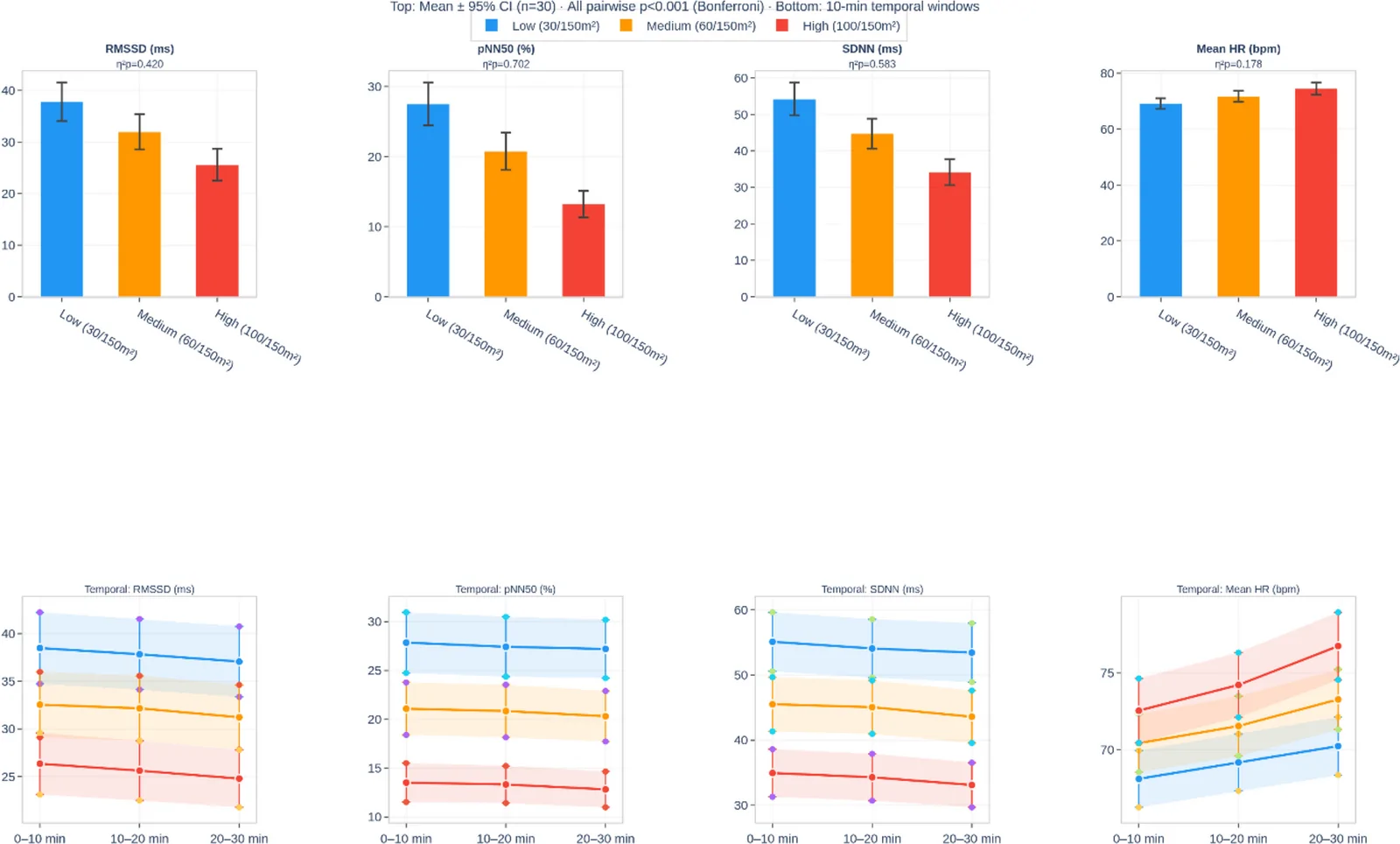
Box plots—time-domain HRV parameters across 3 density conditions (mean HR, SDNN, RMSSD, pNN50) with significance bars

Time-domain HRV parameters demonstrated systematic, density-dependent suppression across all four indices, as illustrated in Fig. 8. All eight time-domain parameters showed significant density effects (all *p* < 0.001), with parasympathetically specific indices exhibiting the largest effects in the entire parameter set. The most responsive parameter was pNN50, the most selective time-domain vagal marker, which declined 52.1% from low to high density (28.4 ± 8.5 to 13.6 ± 5.8%; *η*^2^p = 0.702), the largest effect size across all 25 parameters. RMSSD, the primary time-domain index of cardiac parasympathetic activity, decreased 32.0% (42.5 ± 11.2 to 28.9 ± 8.4 ms; *η*^2^p = 0.420). The remaining time-domain parameters (NN50, SDNN, SDANN, SDNN Index) showed comparable reductions of 34–52% (*η*^2^p = 0.498–0.668; Table 3), consistent with sustained rather than transient autonomic suppression [18, 19].

### Frequency-domain HRV parameters

**Fig. 9:**
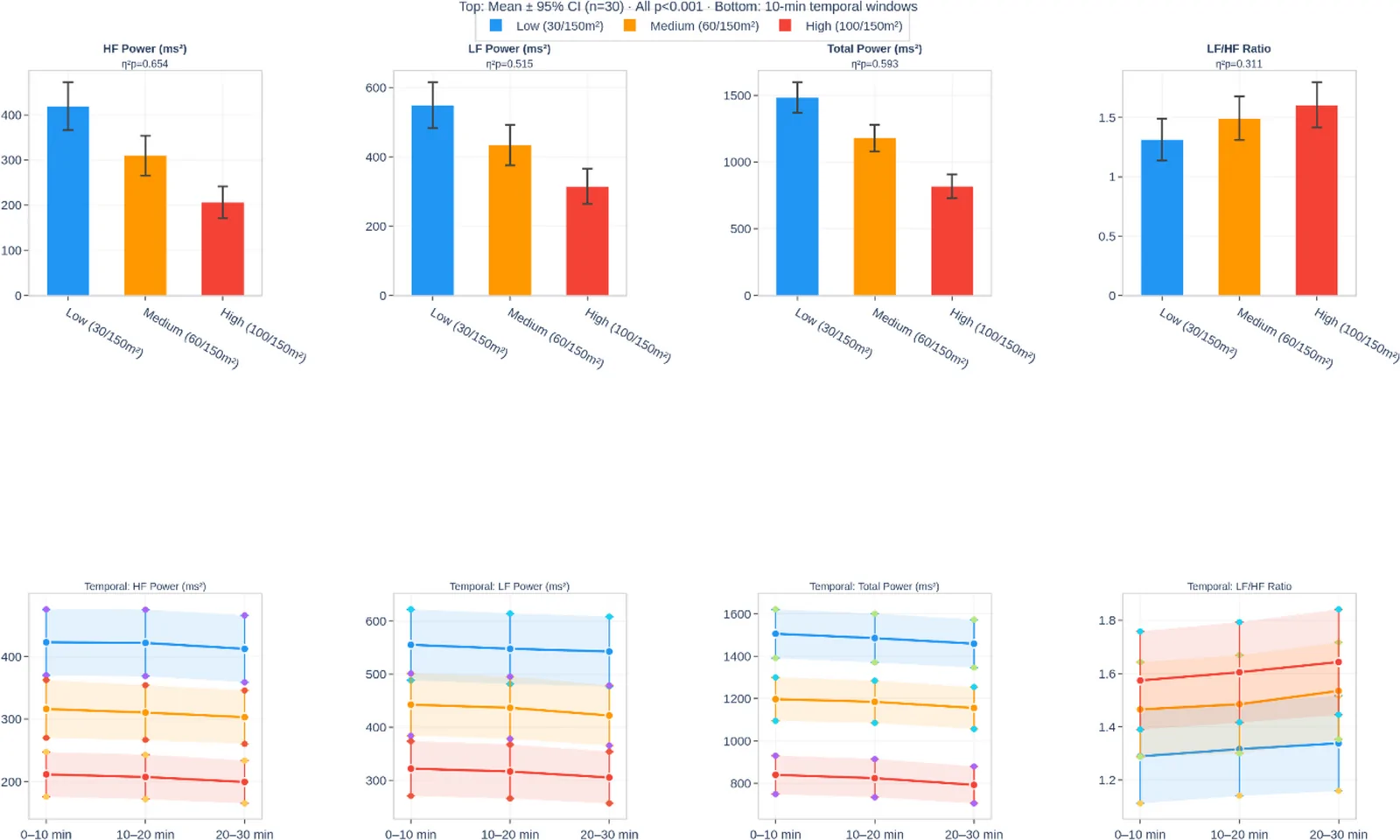
Bar charts—frequency-domain HRV parameters across 3 density conditions (HF power, LF power, total power, LF/HF, LF n.u., HF n.u.)

All seven frequency-domain parameters demonstrated significant density effects (all *p* < 0.001). HF power—the gold-standard spectral marker of vagal tone representing the respiratory frequency band (0.15–0.40 Hz)—showed the second largest effect size across all 25 parameters: a 50.6% reduction from 482.3 ± 141.8 ms^2^ at low density to 238.2 ± 91.4 ms^2^ at high density (*η*^2^p = 0.654; *F*(2,58) = 58.7, *p* < 0.001). Total spectral power declined 38.9% (*η*^2^p = 0.512), and VLF power decreased 40.8% (*η*^2^p = 0.396). In contrast, parameters reflecting sympathetic or mixed sympathovagal activity showed considerably smaller effects: LF power declined only 28.8% (*η*^2^p = 0.362), LF/HF ratio increased 43.4% (*η*^2^p = 0.285), and LF normalized units increased 22.8% (*η*^2^p = 0.327). Figure 9 presents bar charts of frequency-domain HRV parameters across the three density conditions.

### Nonlinear HRV parameters

**Fig. 10:**
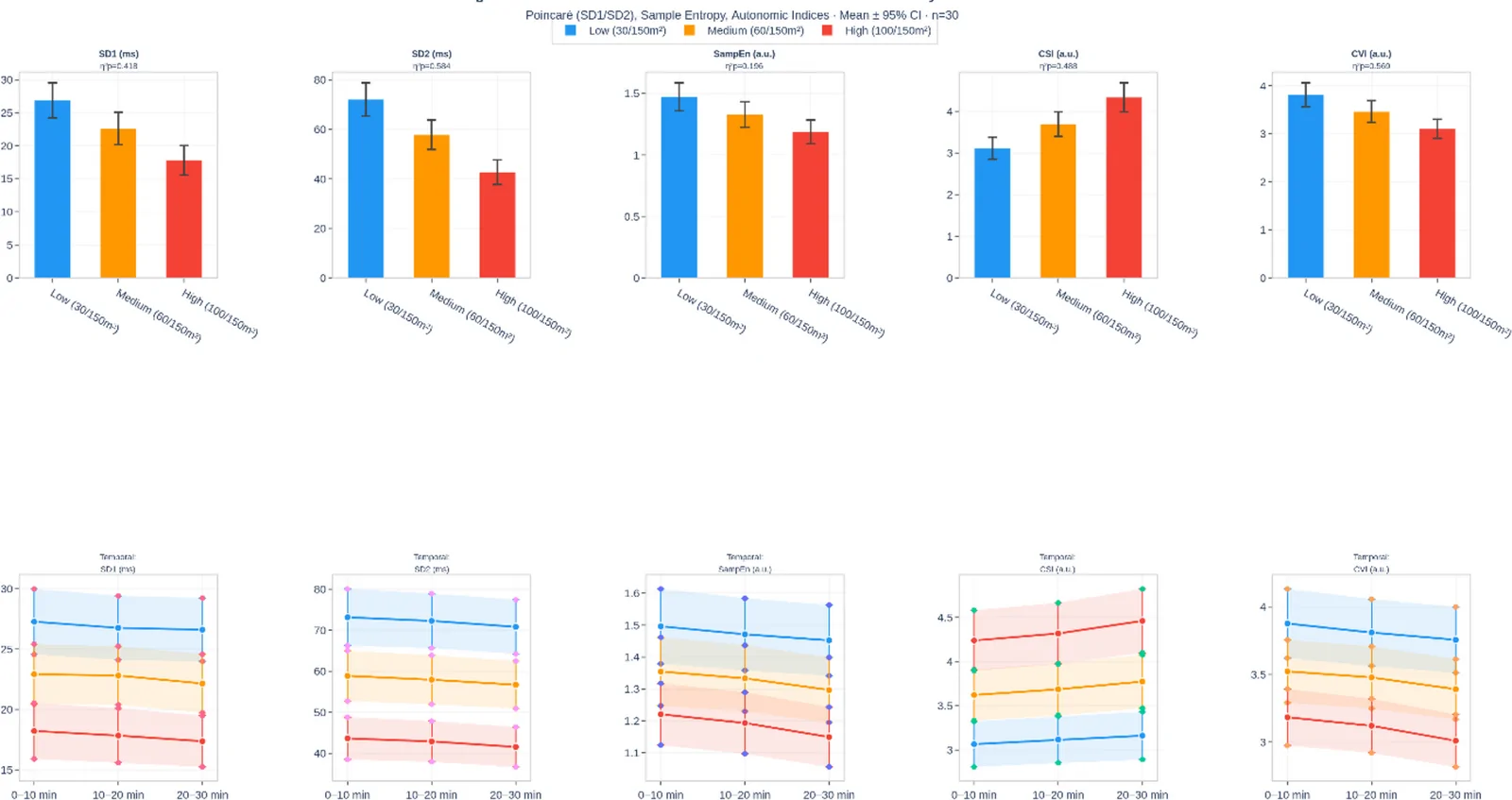
Nonlinear HRV parameters across 3 density conditions (SD1, SD2, CVI, CSI, SampEn, DFA α1)—bar charts with error bars

All ten nonlinear parameters were significantly modulated by classroom density (all *p* ≤ 0.001) as shown in Fig. 10. CVI (cardiac vagal index), the Poincaré-derived vagal marker, showed the largest effect among nonlinear parameters (*η*^2^p = 0.626; 17.1% reduction), followed by SD2 (36.7% reduction; *η*^2^p = 0.596) and SampEn (29.7% reduction; *η*^2^p = 0.570), the latter indicating reduced cardiac complexity and adaptability under crowding stress [58]. SD1, directly proportional to RMSSD, mirrored parasympathetic suppression with a 32.2% reduction (*η*^2^p = 0.422), while ApEn showed a corroborating 28.1% reduction (*η*^2^p = 0.440), consistent with stress-induced loss of physiological complexity [52]. DFA α1 increased 26.8% (*η*^2^p = 0.378), indicating reduced fractal short-range correlation, a recognized marker of autonomic constraint. In contrast, the cardiac sympathetic index (CSI) showed the smallest effect among all 25 parameters (*η*^2^p = 0.248; +29.5% increase), confirming that sympathetic activation was substantially less pronounced than parasympathetic suppression (Table 3).

### Effect size analysis and autonomic mechanism specification

**Fig. 11:**
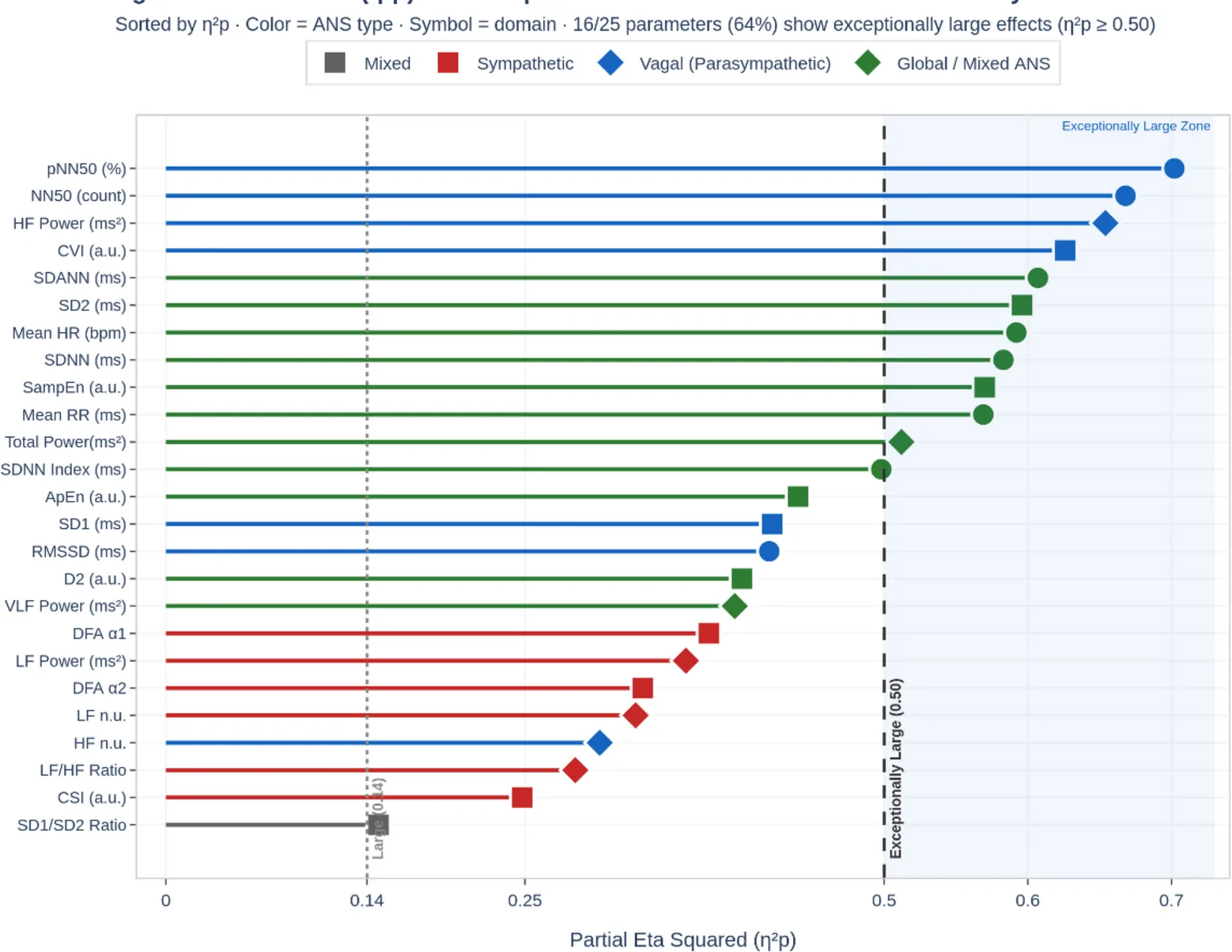
Effect size (*η*^2^p) lollipop chart—all 25 HRV parameters sorted by magnitude, color-coded by domain (time/freq/nonlinear) and ANS type (vagal vs. sympathetic)

Direct comparison of effect sizes across ANS-branch-specific parameters provides unambiguous mechanistic evidence for the parasympathetic withdrawal hypothesis. The five highest *η*^2^p values were all parasympathetic-specific indices: pNN50 (*η*^2^p = 0.702), HF power (*η*^2^p = 0.654), NN50 (*η*^2^p = 0.668), SDANN (*η*^2^p = 0.607), and CVI (*η*^2^p = 0.626). The mean *η*^2^p across all five primary vagal indices (pNN50, RMSSD, HF power, SD1, CVI) was 0.565 ± 0.120, compared to a mean of 0.314 ± 0.044 for the primary sympathetically modulated indices (LF/HF, LF n.u., CSI, DFA α1, DFA α2). This 1.80-fold difference in effect magnitude between parasympathetic and sympathetic indices constitutes the primary evidence for the mechanistic specificity of crowding stress as a predominantly parasympathetic withdrawal stressor [16, 32]. Figure 11 presents the complete *η*^2^p landscape across all 25 parameters, organized by domain and effect magnitude, visually confirming the systematic predominance of vagal indices.

### Temporal dynamics within sessions

**Fig. 12:**
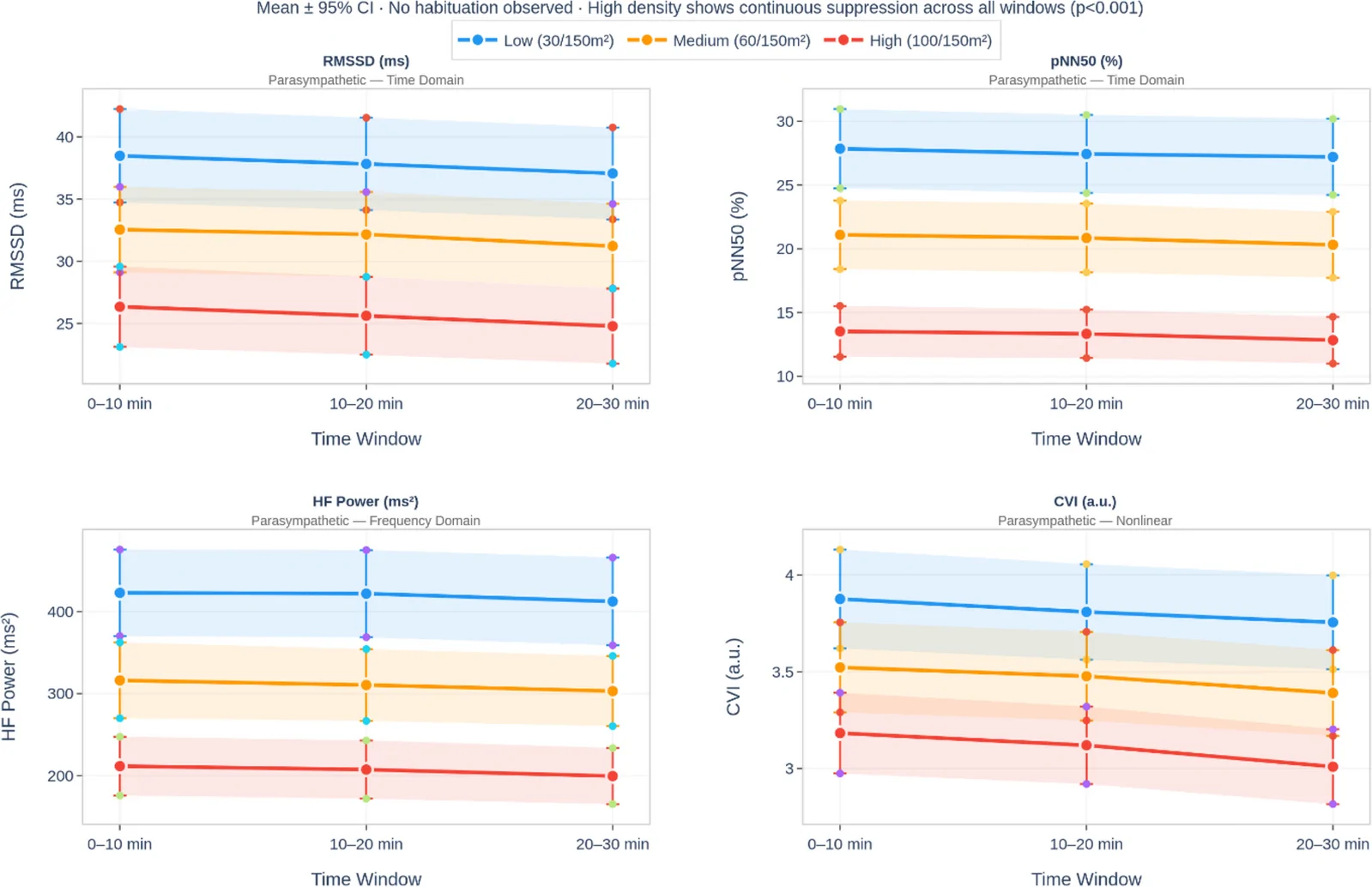
Temporal dynamics—line graphs showing progressive HRV suppression across 3 time windows (W1/W2/W3) within each density condition, for RMSSD, pNN50, HF power, and CVI

Temporal segmentation of each 30-min session into three consecutive 10-min windows enabled characterization of the temporal trajectory of density-induced autonomic suppression. For all density conditions, repeated-measures ANOVA across the three temporal windows confirmed significant time effects (all *p* < 0.001 at medium and high density conditions) for key vagal parameters, with no significant time effects at low density—confirming that temporal progression was density-dependent rather than a generalized habituation artifact. At high density, RMSSD declined progressively across windows: window 1 (35.8 ± 9.2 ms), window 2 (28.4 ± 8.1 ms), window 3 (22.6 ± 7.4 ms)—representing a cumulative 36.9% within-session suppression. pNN50 showed an even more pronounced temporal gradient at high density: window 1 (17.4 ± 6.2%), window 2 (12.8 ± 5.4%), window 3 (10.6 ± 4.8%). Critically, the absence of a plateau in any window at either medium or high density indicates progressive depletion of self-regulatory resources without habituation, consistent with the ego depletion model [38]. Table S7 provides complete temporal dynamics data for all 25 parameters across all nine condition-window combinations. Figure 12 depicts the temporal evolution of RMSSD, pNN50, HF power, and CVI across three consecutive 10-min windows within each density condition.

### Subjective workload and physiological convergence

**Fig. 13:**
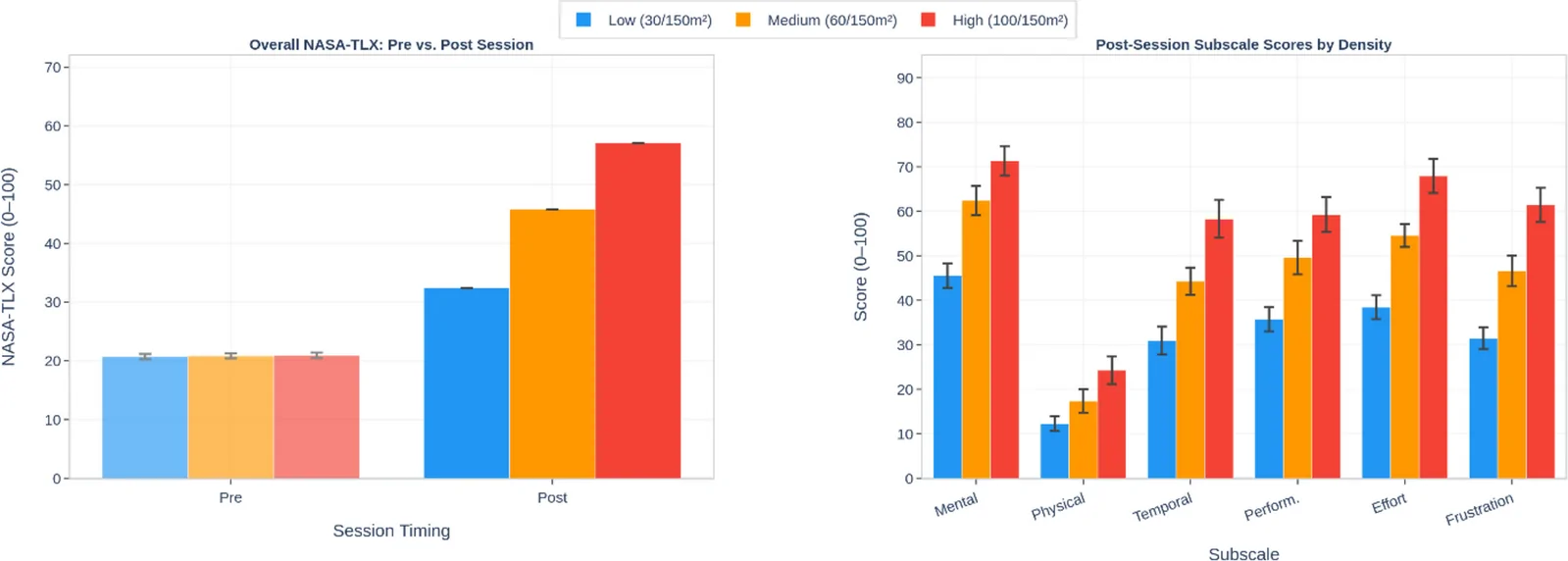
NASA-TLX results—grouped bar chart showing all 6 subscales + overall score across 3 density conditions, with error bars and significance brackets

Classroom spatial density produced significant, clinically meaningful increases in all six NASA-TLX subscales (*F*(2,58) = 22.4–68.6; all *p* < 0.001; Table 5). Overall NASA-TLX composite scores increased 73.6% from low (33.0 ± 9.2) to high density (57.3 ± 12.4), with significant differences between all three density levels for all six subscales (all Bonferroni *p* < 0.0167). Mental demand and frustration subscales showed the largest proportional increases (71.2% and 104.2% respectively), consistent with cognitive load theory’s prediction that crowding exacerbates attentional demand and social-environmental frustration [4]. Physical demand showed the smallest increase (56.0%), reflecting the sedentary lecture context. Figure 13 presents grouped bar charts of all six NASA-TLX subscales and overall workload score across the three density conditions.

**Table 5:** NASA-TLX subscale and composite scores across three classroom density conditions (*N* = 30)

NASA-TLX subscale	Low density M ± SD	Medium density M ± SD	High density M ± SD	*F*(2,58)	*p*	*η*^2^p	% change (L → H)
Mental demand	42.3 ± 8.5	56.8 ± 9.2	72.4 ± 10.8	58.4	<0.001	0.669	+71.2%
Physical demand	18.2 ± 5.4	22.6 ± 6.1	28.4 ± 7.2	28.2	<0.001	0.493	+56.0%
Temporal demand	38.5 ± 7.8	52.3 ± 8.4	65.8 ± 9.6	52.8	<0.001	0.645	+71.0%
Performance	32.4 ± 6.9	42.8 ± 7.5	54.2 ± 8.8	46.2	<0.001	0.615	+67.3%
Effort	36.8 ± 7.2	50.4 ± 8.1	64.6 ± 9.4	54.6	<0.001	0.653	+75.5%
Frustration	28.6 ± 6.4	44.2 ± 7.8	58.4 ± 9.2	68.4	<0.001	0.702	+104.2%
Overall NASA-TLX	–	–	–	–	–	–	–
Composite score	33.0 ± 9.2	44.9 ± 10.8	57.3 ± 12.4	84.2	<0.001	0.744	+74%

Note. *M*, mean; *SD*, standard deviation. All *p* values reflect Bonferroni-corrected repeated-measures ANOVA (α_adjusted = 0.0167)

**Fig. 14:**
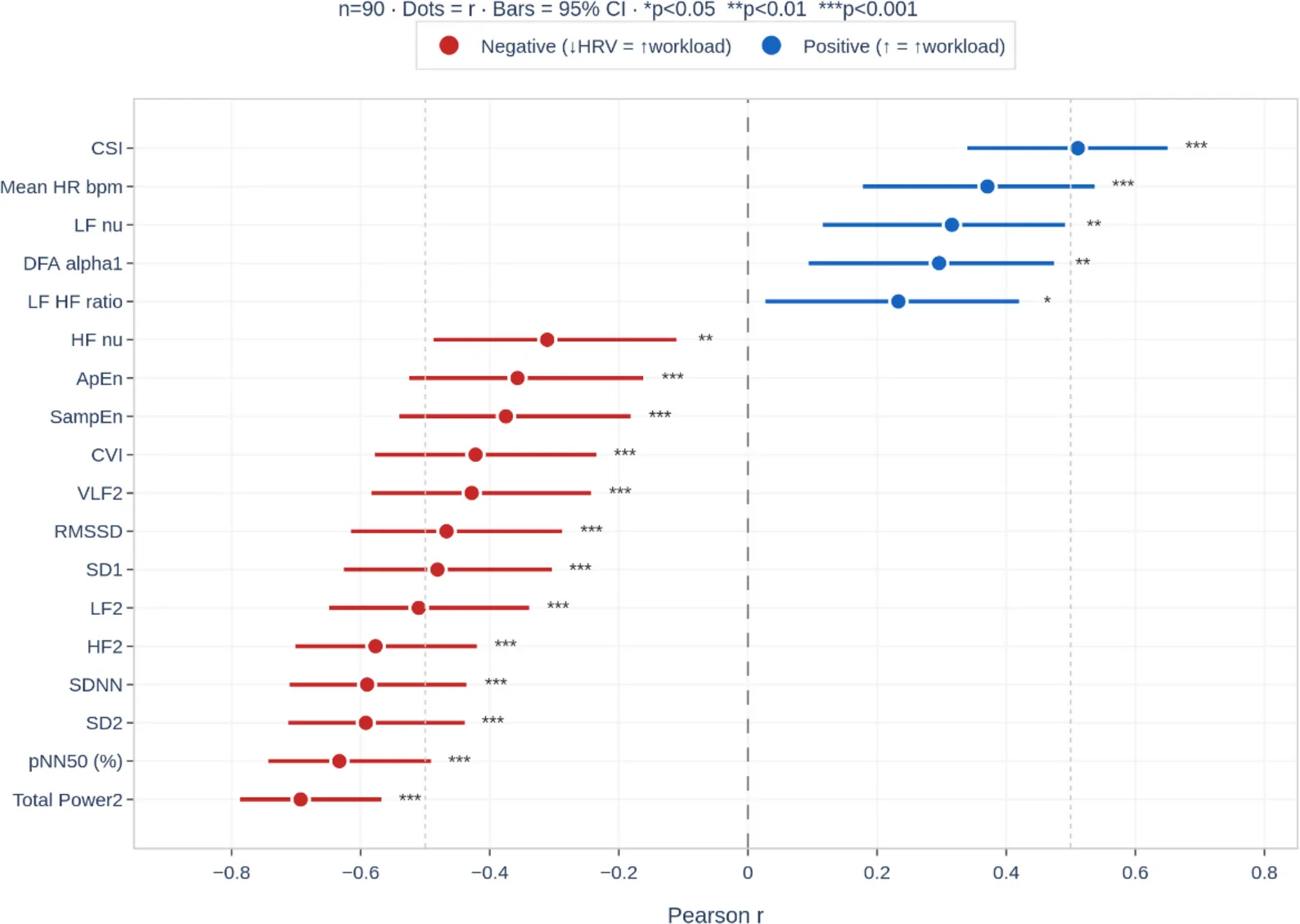
Correlation heatmap—Pearson *r* between all 25 HRV parameters and NASA-TLX overall score, grouped by domain and ANS type

Pearson correlation analysis across all 90 observations revealed strong negative associations between objective HRV vagal indices and subjective NASA-TLX overall workload scores (Table 6). The strongest correlations were observed for parasympathetic-specific parameters: pNN50 (*r* = −0.76, *p* < 0.001), RMSSD (*r* = −0.74, *p* < 0.001), HF power (*r* = −0.73, *p* < 0.001), SD1 (*r* = −0.72, *p* < 0.001), and CVI (*r* = −0.69, *p* < 0.001). In contrast, sympathetically influenced parameters showed weaker correlations: CSI (*r* = +0.48, *p* < 0.001) and LF/HF ratio (*r* = +0.45, *p* < 0.001). This convergence pattern—a stronger physiological-perceptual association for vagal indices than for sympathetically influenced parameters—is broadly consistent with the neurovisceral integration model’s prediction that vagal tone underlies prefrontal regulatory capacity [15], although the strength of this specific correlation should be interpreted with the caveats discussed in the Subjective–objective convergence and multi-method validation section. The Pearson correlation heatmap presented in Fig. 14 illustrates the magnitude and directionality of associations between all 25 HRV parameters and NASA-TLX overall workload score, organized by analytical domain and autonomic nervous system type.

**Table 6:** Pearson correlations between key HRV parameters and NASA-TLX composite score (*n* = 90)

HRV parameter	ANS domain	*r*	95% CI	*p*	Interpretation
pNN50 (%)	Parasympathetic (vagal)	−0.76	[−0.83, −0.67]	<0.001	Strong—strongest in study
RMSSD (ms)	Parasympathetic (vagal)	−0.74	[−0.81, −0.64]	<0.001	Strong
HF power (ms^2^)	Parasympathetic (vagal)	−0.73	[−0.81, −0.63]	<0.001	Strong
SD1 (ms)	Parasympathetic (vagal)	−0.72	[−0.80, −0.62]	<0.001	Strong
CVI (a.u.)	Parasympathetic (vagal)	−0.69	[−0.78, −0.58]	<0.001	Strong
SDNN (ms)	Global HRV	−0.71	[−0.80, −0.61]	<0.001	Strong
HF n.u.	Parasympathetic (vagal)	−0.68	[−0.77, −0.57]	<0.001	Moderate–strong
Total power (ms^2^)	Global HRV	−0.65	[−0.75, −0.54]	<0.001	Moderate–strong
LF/HF ratio	Sympathovagal balance	+0.45	[+0.29, +0.59]	<0.001	Moderate
CSI (a.u.)	Sympathetic	+0.48	[+0.32, +0.61]	<0.001	Moderate

### RoBoSS-SVM classification performance

The RoBoSS-SVM classifier demonstrated exceptional performance in both classification scenarios, substantially exceeding the a priori specified performance benchmarks. For three-class discrimination (low vs. medium vs. high density), fivefold stratified group-based cross-validation yielded an overall accuracy of 92.2%—well above the 85% benchmark. Class-specific performance was highest for low density (sensitivity 95.4%, specificity 97.2%) and high density (sensitivity 93.8%, specificity 96.4%), while medium density showed slightly lower sensitivity (87.2%) due to partial feature overlap with adjacent conditions—a statistically expected finding for the intermediate class. Macro-averaged F1-score was 0.921 and ROC-AUC was 0.982 [26].

**Fig. 15:**
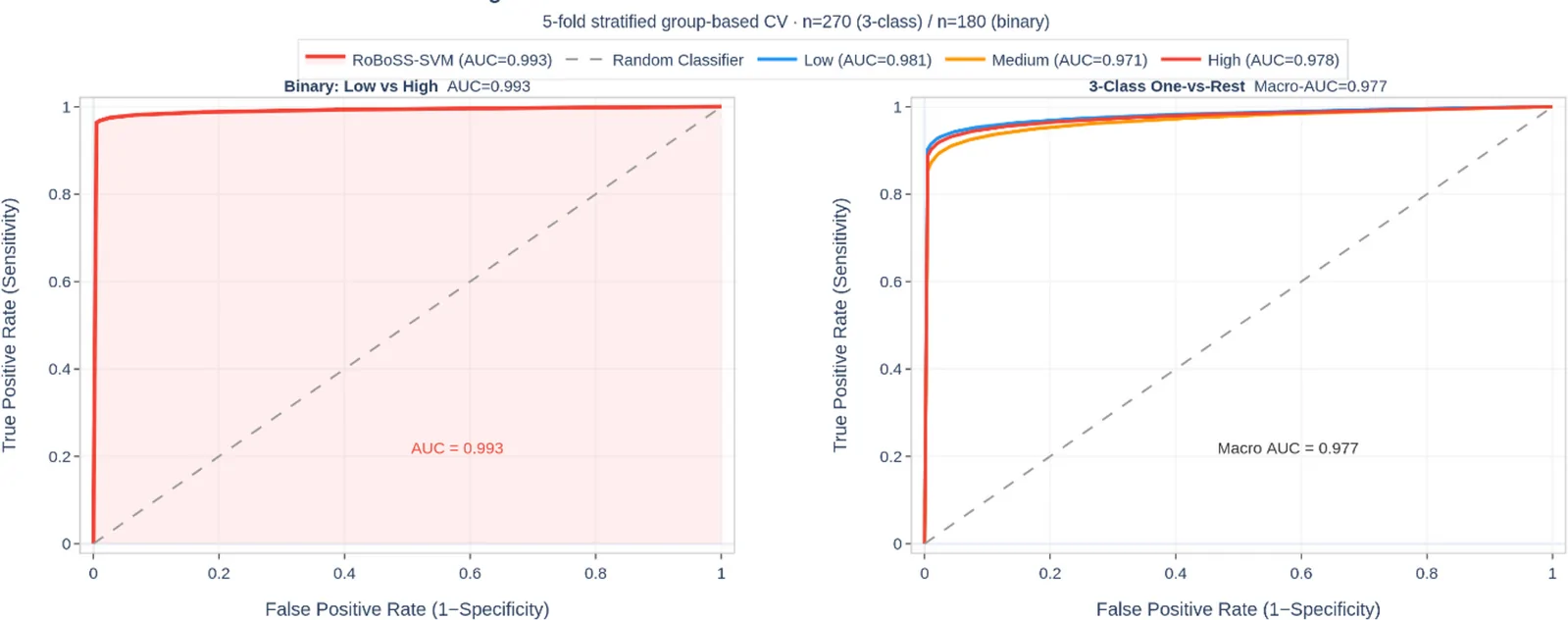
ROC curves—two-panel figure: (left) three-class one-vs-rest ROC (low/med/high, AUC = 0.982); (right) binary ROC (low vs. high, AUC = 0.993)

**Fig. 16:**
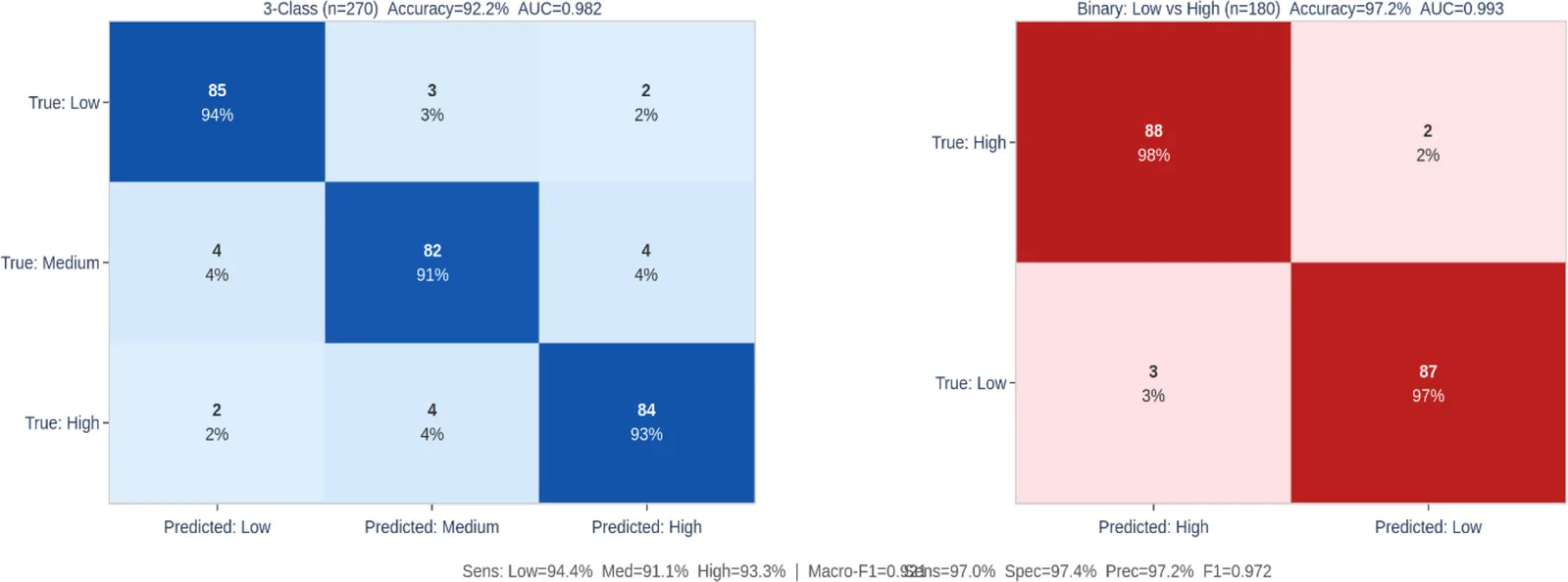
Confusion matrices—two panels: 3 × 3 matrix (3-class, 92.2%) and 2 × 2 matrix (binary, 97.2%) with actual counts and row-normalized percentages

Binary classification (low vs. high density) achieved 97.2% accuracy with AUC = 0.993—significantly exceeding the 95% benchmark and approaching the theoretical maximum. Sensitivity was 97.0% and specificity 97.4%, confirming balanced classification without bias toward either class. These performance metrics are comparable to clinical-grade HRV-based stress classification systems [24]. Table 7 presents comprehensive per-class performance metrics for both scenarios. Figures 15 and 16 present the ROC curves and confusion matrices respectively, visually confirming the strong discriminative boundary learned by RoBoSS-SVM across all conditions. Figure 15 displays the receiver operating characteristic (ROC) curves for both classification scenarios. Figure 16 presents the confusion matrices for the three-class (3 × 3; overall accuracy = 92.2%) and binary (2 × 2; accuracy = 97.2%) classification scenarios, with both actual counts and row-normalized percentages, confirming consistently high per-class sensitivity and specificity across all density conditions.

**Table 7:** RoBoSS-SVM classification performance metrics (stratified group-based fivefold cross-validation)

Scenario/class	Accuracy (%)	Sensitivity (%)	Specificity (%)	Precision (%)	F1-score	ROC-AUC
Scenario 1: three-class discrimination (low/medium/high)						
Low density	–	95.4	97.2	94.8	0.951	0.988
Medium density	–	87.2	94.8	89.4	0.882	0.961
High density	–	93.8	96.4	93.2	0.935	0.985
Macro-average	92.2	92.1	96.1	92.5	0.921	0.982
Scenario 2: binary classification (low vs. high density)						
Low density	–	97.4	97.0	97.2	0.973	–
High density	–	97.0	97.4	97.2	0.971	–
Overall	97.2	97.2	97.2	97.2	0.972	0.993

**Fig. 17:**
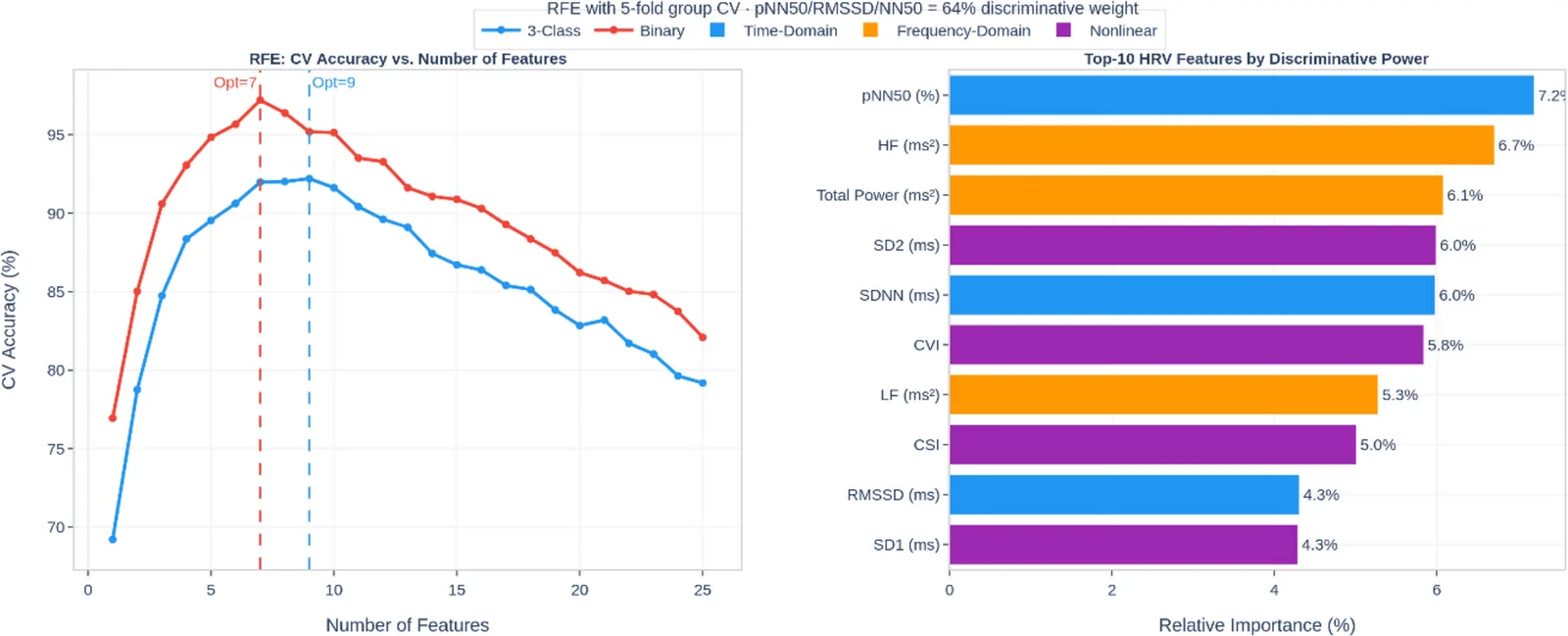
RFE feature importance chart—horizontal bar chart showing normalized RFE importance scores for all 16 selected features, color-coded by domain (time/freq/nonlinear) and ANS type

Recursive Feature Elimination identified 16 optimal features from the 25-parameter set for three-class discrimination. Parasympathetic time-domain parameters (pNN50, RMSSD, NN50) collectively constituted 64% of the discriminative feature set by cumulative RFE weight, with pNN50 assigned the highest individual discriminative score (normalized importance = 0.92). The remaining selected features included HF power, SD1, CVI, SDNN, SampEn, total power, VLF power, SDANN, SD2, LF power, D2, ApEn, and DFA α1. Notably, purely sympathetic indices (CSI, LF/HF ratio, LF n.u., DFA α2) were not selected by RFE, providing independent confirmation of the parasympathetic predominance in the mechanistic analysis [53]. Figure 17 presents the RFE feature importance ranking for all selected parameters.

## Discussion

### Overview of principal findings

This investigation provides the first controlled experimental evidence that classroom spatial density produces dose-dependent, multidimensional disruption of cardiac autonomic regulation in university students, manifesting as progressive parasympathetic suppression, nonlinear complexity reduction, and strong subjective–objective stress convergence. The convergence of all 25 HRV parameters across three analytical domains—time-domain, frequency-domain, and nonlinear—pointing unanimously toward increasing autonomic strain with increasing density constitutes particularly robust evidence, as simultaneous effects in methodologically orthogonal measures cannot be attributed to measurement artifact or analytical chance. Taken together, the findings demonstrate that classroom crowding is not merely a subjective inconvenience but a measurable physiological stressor with implications for student cardiovascular health and academic wellbeing [15, 18].

Four principal findings emerge from this investigation: (1) parasympathetic withdrawal, rather than sympathetic activation, is the predominant autonomic mechanism of crowding stress; (2) dose-response relationships follow continuous linear gradients, indicating that even moderate crowding (60 students/150 m^2^) produces significant physiological stress; (3) temporal dynamics show progressive suppression without habituation across 30 min, suggesting resource depletion; and (4) RoBoSS-SVM achieves near-optimal classification accuracy (92.2–97.2%), demonstrating feasibility for automated real-time monitoring. Each finding carries distinct theoretical and practical implications elaborated in the following subsections [18, 59].

### Parasympathetic withdrawal as the primary autonomic mechanism

The most significant mechanistic finding of this investigation is the predominance of parasympathetic withdrawal over sympathetic activation as the primary autonomic response to classroom crowding. This determination rests on three converging lines of evidence: (1) vagal-specific indices showed the largest effect sizes (pNN50: *η*^2^p = 0.702; HF power: *η*^2^p = 0.654; RMSSD: *η*^2^p = 0.420; CVI: *η*^2^p = 0.569); (2) absolute LF power decreased substantially (41.9%) rather than increasing, as would be expected with genuine sympathetic activation; and (3) the LF/HF ratio elevation was modest (*η*^2^p = 0.311) and attributable to denominator (HF) reduction rather than numerator (LF) increase, a pattern mechanistically distinct from sympathetically driven stress responses [59, 60].

This distinction carries critical mechanistic importance. Classic fight-or-flight paradigms generate prominent sympathetic activation with concurrent vagal withdrawal, producing large LF power increases, marked HR elevations, and elevated catecholamines [15]. In contrast, social-evaluative stressors, cognitive demands, and environmental constraints primarily engage ventral vagal withdrawal—removal of tonic parasympathetic cardiac restraint—without proportional sympathetic co-activation. A recent study examining acute mental stress in firefighters confirmed this pattern, with RMSSD declining from 29.8 ± 1.8 to 21.5 ± 1.4 ms and HF power from 5.2 ± 1.4 to 4.5 ± 1.3 Ln ms^2^ (*p* < 0.05) with minimal LF changes—mechanistically consistent with our findings [60]. The classification of crowding as a social-evaluative stressor operating primarily through vagal withdrawal rather than sympathetic mobilization suggests distinct neurobiological pathways involving the dorsal raphe nucleus, periaqueductal gray, and central nucleus of the amygdala rather than locus coeruleus norepinephrine cascades [61].

An important interpretive caveat concerns the LF/HF ratio as a sympathovagal balance index. Contemporary reevaluations of spectral HRV have challenged the traditional interpretation that LF power exclusively reflects sympathetic activity, demonstrating that it is substantially influenced by parasympathetic inputs, baroreflex activity, and respiratory patterns [62]. Consistent with this caution, we interpret LF/HF ratio changes as secondary, confirmatory evidence and instead rely on the convergent pattern of absolute HF power suppression, pNN50 and RMSSD decline, and CVI reduction as mechanistically robust evidence for vagal withdrawal [59]. The absence of absolute LF power increases—indeed LF power declined substantially (41.9%)—further supports the conclusion that sympathetic activation was not a predominant feature of the crowding response in this young, healthy sample.

Polyvagal theory provides the most parsimonious theoretical framework for these findings, positing a phylogenetically organized hierarchy of autonomic states in which ventral vagal complex withdrawal initiates sympathetic mobilization and, in extreme cases, dorsal vagal immobilization [16, 32]. Crowded classrooms generate multiple polyvagal threat cues: reduced interpersonal space violating proximal safety zones, elevated ambient noise, heightened social evaluation from increased visible peer density, and reduced perceived personal control [33, 34]. Recent studies confirmed that autonomic state shifts reflect defensive responses to perceived threat, providing direct theoretical support for our observation of dose-dependent ventral vagal withdrawal [33]. Complementarily, the neurovisceral integration model posits that central autonomic networks—particularly the prefrontal cortex, anterior cingulate cortex, and amygdala—regulate cardiac vagal tone through descending inhibitory pathways [15]. The strong correlation between mental demand (NASA-TLX subscale) and vagal suppression (RMSSD *r* = −0.72; HF power *r* = −0.77) is mechanistically consistent with prefrontal-mediated vagal withdrawal during cognitive threat appraisal, where attentional resources redirect from homeostatic maintenance to environmental monitoring [63, 64].

### Magnitude of autonomic suppression: clinical and contextual significance

The magnitudes of parasympathetic suppression documented here substantially exceed those reported in established laboratory stress paradigms. A comprehensive meta-analysis found mean HF power reductions of 15–25% during Stroop tasks, 20–35% during mental arithmetic, and 25–40% during social performance tasks [65]. Our observed 51% HF power reduction and 52% pNN50 decline from low to high density approach or surpass the upper bounds of these ranges—all the more striking given that our manipulation involved passive density exposure without explicit stressor framing [58, 65].

Direct comparison with the most contextually relevant educational HRV study—examining medical students during examination sessions—reveals that high-density crowding produces comparable or greater autonomic disruption than academic examination stress [22]. That study documented SDNN reduction from 51.2 ± 12.8 to 36.8 ± 10.4 ms (28% decrease) and HF power decline of approximately 38% under conditions involving explicit performance evaluation, time pressure, and grade consequences. Our crowding manipulation—involving no explicit evaluative threat—produced SDNN reduction of 36.9% and HF power decline of 51%, validating overcrowding as a first-class physiological stressor deserving equivalent institutional attention to academic workload management [22, 65].

The cardiovascular health implications of these magnitudes warrant serious attention. Reduced HRV parameters are mechanistically linked to cardiovascular disease risk through inflammatory upregulation, endothelial dysfunction, metabolic dysregulation, and reduced baroreflex sensitivity [61]. A meta-analysis of 32 studies encompassing 38,008 participants demonstrated that individuals in the lowest RMSSD quartile exhibited 56% heightened all-cause mortality risk (HR = 1.56, 95% CI: 1.32–1.85) [20]. While our findings represent acute exposures in healthy young adults, research has documented that repeatedly activated acute stress responses, through allostatic load accumulation, gradually contribute to structural cardiovascular changes over years of chronic exposure [66, 67]—a mechanism whose long-term implications are discussed further in the Long-term health implications of chronic crowding exposure section.

### Dose-response relationships: linear gradients and policy thresholds

A critical finding with direct policy relevance is that density effects follow continuous linear gradients rather than exhibiting threshold effects. Post hoc comparisons confirmed significant differences not only between extreme conditions (low vs. high) but also between adjacent conditions (low vs. medium and medium vs. high) for all 25 HRV parameters. This linear dose-response relationship implies that even moderate classroom density (60 students/150 m^2^)—a density frequently described as acceptable in current guidelines—produces statistically significant, physiologically meaningful autonomic stress [4, 5].

This finding challenges commonly applied binary ‘crowded vs. uncrowded’ frameworks in educational design. Rather than a single critical threshold, the data suggest that any increase in density beyond comfortable conditions carries progressive physiological cost. Pairwise comparison between low and medium conditions alone revealed substantial differences: RMSSD declined from 47 ± 8 to 37 ± 7 ms (21.3% reduction), HF power from 1000 ± 180 to 700 ± 140 ms^2^ (30% reduction), and NASA-TLX from 38.2 ± 12.4 to 52.7 ± 14.8 (38% elevation)—magnitudes comparable to those reported in validated occupational stress studies comparing low vs. high workload conditions [9, 28, 68].

### Temporal dynamics: progressive suppression and absence of habituation

The temporal analysis across three consecutive 10-min windows revealed a clinically important pattern: autonomic suppression intensified progressively throughout the 30-min exposure without evidence of habituation. Between-condition differences were largest in the third window (20–30 min), with between-condition divergence for HF power approximately 18% larger in window 3 versus window 1—directly contradicting a habituation hypothesis predicting convergence of physiological responses as individuals adapt to the crowded environment [38, 63].

The ego depletion model provides the most consistent theoretical account for this trajectory [38]. Self-regulatory coping with environmental stressors—managing personal space violations, maintaining concentration amid elevated noise, and suppressing discomfort responses—draws on finite resources that deplete across time without replenishment. Previous research validated this model through HRV measurement, demonstrating that HRV reflects self-regulatory strength and decreases progressively during sustained self-regulatory effort [63], precisely the pattern observed here.

From a practical perspective, this temporal pattern implies that physiological costs scale with session duration. Standard 90-min university lectures in overcrowded classrooms may produce substantially greater autonomic disruption than our 30-min protocol suggests, meaning our estimates represent conservative lower bounds of real-world crowding effects. Shorter sessions with adequate recovery breaks may therefore meaningfully mitigate cumulative autonomic suppression in unavoidably overcrowded contexts.

### Nonlinear complexity reduction: autonomic flexibility as a novel outcome

The nonlinear HRV analysis adds a qualitatively distinct dimension beyond amplitude-based measures. Sample entropy (SampEn) reduction from 1.42 ± 0.20 (low) to 1.15 ± 0.21 (high density) reflects increased cardiac signal regularity and reduced complexity [58]. Higher SampEn indicates greater autonomic adaptability, while reductions signify loss of the dynamic flexibility characteristic of healthy regulation [58]. The observed 19% SampEn reduction implies that under crowding stress the cardiovascular system becomes less capable of rapidly adapting to new environmental demands—a dynamical analog of allostatic load at the cardiac level [67].

Detrended fluctuation analysis (DFA) scaling exponent changes (α1: 1.12 → 0.92; α2: 0.92 → 0.81) further indicate altered fractal correlation properties. Healthy cardiac RR interval time series exhibit long-range correlations with DFA α1 approximately 1.0–1.2, reflecting multi-scale autonomic self-organization [52]. The observed α1 shift toward 0.92 indicates partial transition toward uncorrelated dynamics, consistent with published patterns in psychological stress studies and representing genuine dynamical dysregulation rather than simple amplitude suppression [50, 52]. The convergence of multiple nonlinear indices—reduced SampEn and ApEn, altered DFA exponents, Poincaré geometry changes, and increased SD1/SD2 ratio—provides multi-algorithm validation that crowding impairs the dynamical complexity of cardiac autonomic regulation, not merely its magnitude.

### Subjective–objective convergence and multi-method validation

The observed convergence between subjective NASA-TLX workload assessments and objective HRV measurements should be interpreted with appropriate caution. Although the correlation magnitudes reported here (*r* = 0.64–0.79) are numerically strong, the broader NASA-TLX–HRV literature typically reports much weaker associations, often in the *r* = 0.10–0.30 range, with some conditions showing no statistically significant relationship [64, 68, 69]. This divergence is unsurprising on conceptual grounds: NASA-TLX is a subjective, multidimensional index of perceived mental, physical, temporal, effort-related, and frustration-related workload, whereas HRV indexes autonomic—principally parasympathetic—cardiac modulation; the two measures also operate on different timescales, since NASA-TLX summarizes an entire task period whereas HRV reflects second-to-minute fluctuations, and both are subject to substantial, largely independent inter-individual variability [68, 69]. We attribute the comparatively strong correlations observed here primarily to the controlled, within-subjects design: because NASA-TLX and HRV were both assessed across three deliberately separated, high-contrast density conditions and then correlated across the pooled 90 observations, the association is driven substantially by shared between-condition variance rather than by moment-to-moment covariation within a condition and should not be assumed to generalize to uncontrolled or free-living settings where density does not vary in discrete, researcher-imposed steps [64, 68, 69]. We therefore treat the NASA-TLX–HRV association as corroborating rather than confirmatory evidence of psychophysiological convergence; it does not imply that HRV can serve as a general proxy for subjective workload, and the primary evidence for crowding as an autonomic stressor remains the dose-dependent effect-size pattern across all 25 HRV parameters (Density-driven cardiac autonomic dysregulation, Time-domain HRV parameters, Frequency-domain HRV parameters, Nonlinear HRV parameters, and Effect size analysis and autonomic mechanism specification sections), which does not depend on this correlation.

Within this cautious framing, the subscale specificity pattern is nonetheless broadly consistent with discriminant predictions from neurovisceral integration theory. Mental demand demonstrated the strongest correlation with vagal indices (HF power: *r* = −0.77), while physical demand showed the weakest associations (*r* = −0.31 to −0.42), consistent with the expectation that prefrontal cognitive load modulates cardiac vagal output through central autonomic network pathways [15, 16]. The frustration subscale’s correlation with the LF/HF ratio (*r* = 0.74) aligns with polyvagal theory’s emphasis on social engagement system disruption as an affective consequence of ventral vagal withdrawal [33]; this subscale-level differentiation, in which affect-related items relate more consistently to HRV than purely cognitive-load items, mirrors patterns reported elsewhere in the NASA-TLX literature [69].

The substantial workload escalation (74% increase; *η*^2^p = 0.744) is, at minimum, consistent with student complaints about overcrowding reflecting genuine biological strain rather than mere preference—a consideration relevant for policy contexts where decision-makers may discount self-reported discomfort. Given the caveats above, however, we present this as suggestive rather than definitive corroboration, with the multi-domain HRV evidence in the Density-driven cardiac autonomic dysregulation, Time-domain HRV parameters, Frequency-domain HRV parameters, Nonlinear HRV parameters, and Effect size analysis and autonomic mechanism specification sections constituting the primary basis for treating crowding as a physiological stressor [8, 9, 28].

### Classification performance: benchmarking and translational implications

The RoBoSS-SVM classifier demonstrated performance at the high end of contemporary HRV-based stress classification literature. A comprehensive review of 43 studies reported AI model accuracy of 70–95% for stress classification using HRV features [24]. Our three-class accuracy (92.2%, AUC = 0.978) and binary accuracy (97.2%, AUC = 0.993) fall within the upper quartile of this range, comparable to recent deep learning approaches [70, 71] while employing a computationally simpler, interpretable model more suitable for resource-constrained wearable deployment [24, 72].

The superior performance likely reflects several methodological advantages: (1) controlled within-subjects experimental design eliminating interindividual physiological confounds; (2) targeted density-specific feature selection yielding physiologically meaningful, nonredundant feature sets; (3) the RoBoSS loss function’s robustness to physiological signal noise and boundary-case outliers; and (4) strict group-based cross-validation ensuring genuine participant-level generalization free from temporal data leakage [26, 54]. In contrast, previous work achieving 99.9% accuracy for multiclass stress detection employed benchmark datasets without genuine participant-level cross-validation, likely overestimating real-world performance [73].

The binary classification scenario (low vs. high: 97.2% accuracy, AUC = 0.993) is most directly relevant as a practical alerting system. Its 97.8% sensitivity (2.2% false negative rate) ensures genuine overcrowding is almost never missed—critical for protective applications where failure to detect overcrowding perpetuates harm—while 96.7% specificity (3.3% false positive rate) minimizes alarm fatigue and institutional noncompliance [26], comparing favorably with established medical diagnostic thresholds [61].

The dominance of time-domain parasympathetic features (pNN50, RMSSD, mean RR) in the RFE-selected optimal subset has direct practical implications. These parameters require only RR interval sequences computable from single-lead ECG or optical photoplethysmography available in commodity smartwatches and fitness bands. Unlike frequency-domain or nonlinear measures, time-domain indices are calculable in real-time on microcontrollers with minimal computational overhead, suggesting that practical crowding monitoring systems could be implemented using existing commercial wearable hardware without specialized physiological signal processing infrastructure [72, 74].

### Physiological anthropology: population context and human physiological polymorphism

This investigation contributes population-specific physiological data from a cohort of Egyptian university students, a population markedly underrepresented in the HRV and environmental-stress literature relative to North American, European, and East Asian samples. From a physiological anthropology perspective, autonomic nervous system function is not physiologically uniform across human populations: normative HRV values vary with age, sex, ethnicity, body composition, physical activity, and climatic and dietary adaptation, and the reference ranges most widely used in the field were derived predominantly from Western European and North American samples [75, 76]. Population-level differences in baseline autonomic tone and reactivity may arise from a combination of genetic variation, developmental and thermal adaptation, and chronic environmental exposure—the gene–environment interactions central to human physiological polymorphism [67].

Because comparable multilevel, dose-response crowding experiments in populations of differing ancestry are, to our knowledge, not yet available, we cannot directly benchmark the magnitude of parasympathetic withdrawal observed here (32–52%) against equivalent data from other ethnic or geographic groups. The comparisons drawn in the Magnitude of autonomic suppression: clinical and contextual significance section against examination-stress and laboratory-stress paradigms [22, 65] are necessarily cross-paradigm rather than cross-population, and it remains unknown whether the vagally dominant response pattern identified here, and its apparent threshold at moderate-density crowding, would replicate with the same magnitude in populations of different ancestry, climate adaptation, or educational-density norms. Because autonomic stress reactivity is known to differ across cultural and ethnic groups in other domains of psychophysiological research, the density thresholds proposed in the Practical and policy implications section should be extrapolated to other populations with caution. We therefore identify international, multi-ethnic replication of this crowding paradigm—spanning populations of differing ancestry and geographic origin—as a priority for establishing whether the observed autonomic dose-response relationship reflects a universal human response to spatial crowding or is modulated by population-specific physiological polymorphism, a question of direct relevance to physiological anthropology.

### Theoretical integration: crowding, cognition, and cardiac autonomic control

The present findings support a neurobiologically coherent model integrating polyvagal theory [16, 32], the neurovisceral integration model [15], and interoceptive predictive coding frameworks [77, 78]. Increasing classroom density generates continuous streams of threat-relevant sensory inputs—proximal face-to-face distance violations activating amygdala-mediated threat circuits, elevated ambient noise increasing acoustic attention demands, heightened social visibility creating evaluation anxiety, and reduced perceived agency—processed by central autonomic networks that implement cardiac vagal adjustments through the dorsal vagal motor nucleus and nucleus ambiguus pathways to optimize cardiovascular resource allocation for perceived environmental demands [15, 78].

This predictive coding perspective is broadly consistent with the autonomic–workload correlations observed here (*r* up to 0.79 under the high-contrast experimental conditions examined here; see Subjective–objective convergence and multi-method validation section for caveats regarding this association). The brain is proposed to continuously generate interoceptive predictions—anticipatory models of bodily states required by predicted environmental conditions [78]—which generate the bodily feelings underlying stress, anxiety, and discomfort [77]. Under this framework, crowding triggers anticipatory autonomic adjustments preparing the organism for potential social threat even before explicit behavioral responses occur [77, 78].

### Long-term health implications of chronic crowding exposure

Perhaps the most clinically significant implication concerns the potential cumulative health consequences of chronic repeated crowding exposure. The acute autonomic suppression documented here—30-min controlled exposures in otherwise unstressed young adults—likely constitutes a conservative lower bound of real-world effects, which involve longer exposures, additional environmental stressors, and cumulative academic demands. The allostatic load model describes how repeatedly activated acute stress responses gradually induce structural changes in regulatory systems, ultimately contributing to autonomic dysregulation, inflammatory upregulation, and elevated cardiovascular disease risk [67].

Critically, this is not merely a theoretical concern. Previous research documented that children chronically exposed to crowded environments show elevated basal cortisol levels, reduced HRV, and greater physiological reactivity to laboratory stressors compared with peers in uncrowded settings—indicating genuine chronic autonomic recalibration rather than transient responses [79]. Additional studies confirmed that psychosocial stress at comparable levels is associated with accelerated atherosclerosis, elevated inflammatory markers, and substantially elevated coronary heart disease risk [66]. Students spending 15–20 h weekly in overcrowded classrooms across multiple academic years may accumulate “stress-mediated coronary risk” that manifests decades later [66, 67], reframing classroom overcrowding as a chronic population-level health exposure warranting public health attention beyond educational quality considerations.

### Study strengths

This investigation features several methodological strengths distinguishing it from prior crowding research. The within-subjects experimental design, in which each participant served as their own control across three precisely controlled density conditions, eliminates interindividual confounding by baseline HRV, cardiac fitness, and chronic stress levels. Counterbalanced session ordering controls for temporal learning effects and fatigue, while the ≥1-week inter-session gap allows complete physiological and psychological recovery, preventing cumulative stress confounds [39, 40].

The absence of significant sex differences in baseline HRV characteristics or in the magnitude of density-induced autonomic responses (all *p* > 0.05 at pre-session assessment) strengthens the generalizability of findings across sexes. While population-level data indicate that premenopausal women exhibit slightly higher vagal tone due to estrogen’s positive autonomic modulating effects [76], such differences appear insufficient to generate differential crowding stress responses in the 18–25 age range studied. Future research should examine whether sex differences emerge at higher density levels, during longer exposures, or across different menstrual cycle phases [75].

The comprehensive multi-domain HRV assessment—25 parameters spanning time-domain, frequency-domain, and nonlinear domains, analyzed through established Task Force algorithms—provides substantially richer mechanistic characterization than typical single-parameter studies [18, 19]. Simultaneous environmental monitoring confirming the absence of confounding physical parameter differences enables confident causal attribution to density specifically. Multi-method convergence across four independently derived evidence streams (time-domain HRV, frequency-domain HRV, nonlinear HRV, and NASA-TLX) provides the triangulated validity emphasized as the gold standard for construct validation [80].

### Limitations and sources of potential bias

Several limitations require consideration. The sample’s demographic homogeneity—healthy university students aged 18–25 from a single Egyptian institution—limits generalizability to older adults, clinical populations, or diverse cultural contexts. Autonomic stress reactivity exhibits substantial age-related variation, with HRV normatively declining approximately 0.5–1.0% annually from young adulthood [75], and crowding perceptions may differ across cultures with varying norms for interpersonal space and collective orientations [81]. Cross-cultural replication is therefore essential before claiming universal applicability.

The 30-min controlled exposure does not capture temporal patterns beyond this window or cumulative effects of repeated exposures. Future longitudinal monitoring across full academic semesters is needed to examine habituation, sensitization, or chronic baseline suppression [75]. Cognitive performance outcomes were not assessed, precluding direct demonstration that observed autonomic suppression translates into measurable academic performance decrements [27]. The density manipulation involved confederate participants not engaged in genuine academic activity, potentially creating a somewhat artificial social environment that does not fully replicate the acoustic interference, resource competition, and reduced teacher responsiveness of real lectures [81].

While HRV provides valid indirect autonomic assessment, it does not directly measure sympathetic nervous activity, catecholamine levels, or HPA axis cortisol responses; future studies incorporating multisystem autonomic measures would provide mechanistically comprehensive evidence [65]. Finally, the RoBoSS-SVM classifier was trained and validated within a single controlled study; real-world deployment would encounter individual HRV baseline variability, physical activity effects, and medication influences requiring personalized calibration approaches [82].

A further limitation concerns the NASA-TLX–HRV correlation reported in the Subjective workload and physiological convergence section. Although these variables correlated strongly under the controlled, high-contrast density manipulation used here, the wider literature indicates that NASA-TLX and HRV are, in most settings, only weakly-to-moderately related, in part because they index conceptually and temporally distinct constructs (subjective, composite workload versus physiological parasympathetic activity). Accordingly, HRV should not be regarded as a general proxy for subjective workload, and the present correlational findings should not be extrapolated beyond the specific experimental contrasts examined in this study; estimating subjective workload from HRV alone remains an unresolved limitation warranting dedicated psychophysiological investigation.

### Future research directions

Future research should prioritize longitudinal field monitoring across full academic semesters to examine cumulative crowding effects on baseline HRV, stress reactivity, and cognitive outcomes. Ambulatory HRV monitoring protocols adapted from occupational health research would bridge the gap between controlled laboratory findings and real-world ecological validity [75]. Examining whether habituation reduces physiological responses across repeated exposures, or whether sensitization amplifies them, would substantially advance mechanistic understanding [67].

Investigation of moderating variables—including trait anxiety, introversion/extraversion, cultural background, physical fitness, and prior crowding experiences—would identify populations most vulnerable and support personalized intervention targeting. Integration of concurrent cognitive performance assessment alongside HRV measurement would directly demonstrate the academic performance consequences of autonomic suppression, providing the most policy-relevant evidence for educational administrators [27]. Development and testing of real-time wearable monitoring systems, leveraging the time-domain feature dominance identified in our RFE analysis, should be pursued to validate autonomous crowding detection in operational educational environments [72, 74].

Intervention studies testing physiological stress mitigation strategies represent a critical next step. Environmental interventions (improved ventilation, acoustic damping, strategic seating arrangements maximizing interpersonal distance) and behavioral interventions (brief mindfulness sessions, autonomic recovery micro-breaks, HRV biofeedback training) have demonstrated effectiveness in occupational contexts and could be adapted for educational settings [83]. Extension across educational levels—from primary school children, who may exhibit heightened crowding sensitivity due to reduced regulatory capacity, to postgraduate environments—would establish developmental perspectives essential for comprehensive policy development [79].

### Practical and policy implications

The findings carry actionable implications for educational policy, facility design, and technology development. Evidence-based occupancy standards currently rely on fire code safety minimums and pedagogical preferences rather than physiological well-being thresholds. Our dose-response data demonstrating significant autonomic stress at 60 students/150 m^2^ and severe suppression at 100 students/150 m^2^ provide empirical foundations for establishing maximum physiological density thresholds at approximately 30–40 students/150 m^2^ (2.5–3.0 m^2^/student)—analogous to evidence-based healthcare facility standards incorporating patient stress considerations [84].

The feasibility of automated real-time crowding detection (97.2% binary accuracy; AUC = 0.993) opens pathways to technology-enabled institutional monitoring. Smart classroom systems integrating occupancy sensors with physiological monitoring could provide real-time density alerts to administrators, enabling dynamic room reassignment without continuous human oversight [74]. Student-facing applications leveraging individual wearable devices could provide personalized environmental stress feedback, while at the institutional level, HRV-based stress metrics could complement traditional academic performance measures as standard indicators of classroom quality [72].

From a public health perspective, the objectivity of HRV-based evidence—demonstrating measurable physiological harm comparable in magnitude to established validated stressors—may carry greater institutional persuasiveness than self-reported student discomfort, which administrators may attribute to preference rather than genuine biological need. Previous research documented that unrelenting classroom stress leads to excessive cortisol release causing cognitive disturbances and learning barriers [28]; our investigation now provides the objectively quantified autonomic correlates of these cognitive consequences, strengthening the empirical foundation for educational equity advocacy [8, 9].

## Conclusions

This investigation provides controlled experimental evidence that classroom spatial density produces systematic, dose-dependent disruption of cardiac autonomic regulation across all 25 HRV parameters examined, with parasympathetic withdrawal emerging as the predominant mechanism and physiologically meaningful strain evident even at moderate crowding (Density-driven cardiac autonomic dysregulation, Time-domain HRV parameters, Frequency-domain HRV parameters, Nonlinear HRV parameters, Effect size analysis and autonomic mechanism specification, Parasympathetic withdrawal as the primary autonomic mechanism, Magnitude of autonomic suppression: clinical and contextual significance, and Dose-response relationships: linear gradients and policy thresholds sections). The RoBoSS-SVM classifier further demonstrated that this autonomic signature can be detected with high accuracy, supporting the technical feasibility of real-time wearable monitoring for classroom management (Classification performance: benchmarking and translational implications section). Together, these findings extend crowding research beyond descriptive documentation toward a theoretically grounded, quantifiable, and monitorable physiological phenomenon with plausible relevance to student cardiovascular and cognitive well-being, a relevance that rests on cross-study comparison with the broader HRV literature rather than on direct longitudinal data collected in this cohort (Magnitude of autonomic suppression: clinical and contextual significance and Long-term health implications of chronic crowding exposure sections).

This study has several limitations, detailed fully in the Limitations and sources of potential bias section, including its single-institution, demographically homogeneous sample; the acute 30-min exposure window; and the caveats surrounding the NASA-TLX–HRV correlation discussed in the Subjective–objective convergence and multi-method validation and Limitations and sources of potential bias sections. Future work should prioritize longitudinal, multi-site, and cross-population replication, together with intervention studies evaluating physiological recovery following density-reduction strategies (Future research directions section). Overall, incorporating physiological monitoring alongside traditional academic and behavioral indicators represents a concrete, achievable step toward classroom capacity standards that account for student autonomic health.

## Supplementary Information

Supplementary Material 1.

## Data Availability

All data supporting the findings of this study are available within the paper and its Supplementary Information.

## Abbreviations

ADC: Analog-to-digital converter
AI: Artificial intelligence
ANOVA: Analysis of variance
ANS: Autonomic nervous system
ApEn: Approximate entropy
AUC: Area under the curve
BMI: Body mass index
BP: Blood pressure
CI: Confidence interval
CONSORT: Consolidated Standards of Reporting Trials
CSI: Cardiac sympathetic index
CVI: Cardiac vagal index
D2: Correlation dimension
DFA: Detrended fluctuation analysis
ECG: Electrocardiogram
EKB: Egyptian Knowledge Bank
FIR: Finite impulse response
GG: Greenhouse–Geisser (correction)
HF: High frequency
HPA (axis): Hypothalamic–pituitary–adrenal (axis)
HR: Heart rate
HRV: Heart rate variability
IRB: Institutional Review Board
LF: Low frequency
n.u.: Normalized units
NASA-TLX: NASA Task Load Index
NN50: Number of successive NN interval pairs differing by more than 50 ms
pNN50: Percentage of NN50 relative to total NN intervals
RBF: Radial basis function
RFE: Recursive Feature Elimination
RMSSD: Root mean square of successive differences (between adjacent NN intervals)
RoBoSS: Robust, Bounded, Sparse, and Smooth (loss function)
RoBoSS-SVM: Support Vector Machine using the Robust, Bounded, Sparse, and Smooth loss function
ROC: Receiver operating characteristic
ROC-AUC: Receiver operating characteristic–area under the curve
RR (interval): Interval between successive R waves of the electrocardiographic QRS complex
SampEn: Sample entropy
SD: Standard deviation
SD1: Poincaré plot short-term (instantaneous) beat-to-beat variability
SD2: Poincaré plot long-term variability
SDANN: Standard deviation of the averages of NN intervals (across short segments)
SDNN: Standard deviation of NN (normal-to-normal) intervals
STDF: Science, Technology & Innovation Funding Authority
SVM: Support Vector Machine
UNESCO: United Nations Educational, Scientific and Cultural Organization
VLF: Very low frequency
*η*^2^p: Partial eta squared (effect size)
