# Supplementary material for: Dose-dependent cardiac autonomic dysregulation under classroom crowding: heart rate variability analysis and machine learning classification
Source: J Physiol Anthropol. 2026 Oct 17;45:23. doi: 10.1186/s40101-026-00451-9 (PMC13675970; doi:10.1186/s40101-026-00451-9)
Supplement: Supplementary file 1 [file 40101_2026_451_MOESM1_ESM.pdf]

Dose-Dependent Cardiac Autonomic Dysregulation Under Classroom Crowding: Heart Rate Variability Analysis and Machine Learning Classification

Table S1. Participant Screening, Enrollment, and Retention Flow (CONSORT-Adapted)

Stage	n	Details / Criteria Applied
**Individuals assessed for eligibility**	58	Campus advertisement respondents
**Excluded at screening**	28	Cardiovascular disease: 3 | Medications affecting ANS: 4 | BMI out of range: 5 | Resting HR abnormal: 2 | Psychiatric disorder: 3 | Smoker: 4 | Unable to attend 3 sessions: 6 | Refused to participate: 1
**Enrolled and randomized**	**30**	Within-subjects; counterbalanced session order
**Completed Session 1 (Low density)**	30	No adverse events; 0 exclusions
**Completed Session 2 (Medium density)**	30	No adverse events; 0 exclusions
**Completed Session 3 (High density)**	30	No adverse events; 0 exclusions
**Segments excluded (signal quality)**	0	All 270 segments passed artifact <5% threshold
**Final analysis dataset**	**30 participants 270 segments**	3 conditions × 3 windows × 30 participants

Note. Green rows = key milestones. ANS = Autonomic Nervous System; BMI = Body Mass Index; HR = Heart Rate. Inter-session interval ≥ 7 days for all participants. References: [42], [41], [45].

Table S2. Definitions, Formulas, and Physiological Interpretations of All 25 HRV Parameters

#	Parameter	Symbol	Unit	Formula / Definition	Physiological Meaning	Ref
	**TIME-DOMAIN PARAMETERS (8)**					
**1**	Mean RR Interval	**Mean RR**	ms	*Mean of all NN intervals*	Overall cardiac rate; inverse of HR	[18]
**2**	Mean Heart Rate	**Mean HR**	bpm	*60,000 / Mean RR*	Tonic cardiac chronotropic state	[18]
**3**	Std Dev NN Intervals	**SDNN**	ms	$\sqrt{[\Sigma(RR_i - MeanRR)^2/(N-1)]}$	Global HRV; total autonomic modulation	[18]
**4**	Root Mean Square Successive Differences	**RMSSD**	ms	$\sqrt{[\Sigma(RR_{i+1} - RR_i)^2/(N-1)]}$	Vagal (parasympathetic) activity; primary short-term index	[18]
**5**	Number of successive NN >50 ms	**NN50**	count	*Count of successive pairs differing by >50 ms*	Parasympathetic modulation; vagal burst sensitivity	[18]
**6**	Proportion of NN50	**pNN50**	%	*NN50 / Total NN × 100*	Most specific time-domain vagal index	[18]
**7**	SD of 5-min NN means	**SDANN**	ms	*SD of mean NN in 5-min epochs*	Long-term HRV; circadian modulation	[18]
**8**	Mean of 5-min NN SDs	**SDNN Index**	ms	*Mean of SDNN in 5-min epochs*	Short-term variability averaged over recording	[18]
	**FREQUENCY-DOMAIN PARAMETERS (7)**					
**9**	Very Low Frequency Power	**VLF**	ms²	*Spectral power 0.003–0.04 Hz (AR, Burg order=16)*	Thermoregulation, vasomotion, renin-angiotensin system	[18],[51]
**10**	Low Frequency Power	**LF**	ms²	*Spectral power 0.04–0.15 Hz*	Baroreflex; mixed sympatho-vagal; partly parasympathetic	[18],[62]
**11**	High Frequency Power	**HF**	ms²	*Spectral power 0.15–0.40 Hz (respiratory band)*	Gold-standard vagal (parasympathetic) spectral marker	[18],[59]
**12**	Total Spectral Power	**Total Power**	ms²	*VLF + LF + HF*	Overall autonomic variability magnitude	[18]
**13**	LF-to-HF Ratio	**LF/HF**	—	*LF (ms²) / HF (ms²)*	Sympathovagal balance (interpret cautiously)	[18],[62]
**14**	LF Normalized Units	**LF (n.u.)**	n.u.	*LF/(Total−VLF)×100*	Relative LF contribution;	[18]

					reduces total power influence	
**15**	HF Normalized Units	**HF (n.u.)**	n.u.	*HF/(Total−VLF)×100*	Relative vagal spectral contribution	[18]
	**NONLINEAR PARAMETERS (10)**					
**16**	Poincaré Short-Axis SD	**SD1**	ms	$RMSSD / \sqrt{2}$	Instantaneous beat-to-beat variability; vagal tone	[52]
**17**	Poincaré Long-Axis SD	**SD2**	ms	$\sqrt{(2 \cdot SDNN^2 - SD1^2/2)}$	Long-term HRV variability; combined ANS activity	[52]
**18**	Poincaré Ratio	**SD1/SD2**	—	*SD1 / SD2*	Balance between short- and long-term variability	[52]
**19**	Sample Entropy	**SampEn**	a.u.	$-\ln(A/B)$; $m=2$, $r=0.2 \cdot SD$	Cardiac complexity; higher = more adaptive	[52],[58]
**20**	Approximate Entropy	**ApEn**	a.u.	$ApEn(m=2, r=0.2 \cdot SDNN, N)$	Regularity measure; less robust than SampEn	[52]
**21**	Correlation Dimension	**D2**	a.u.	*Grassberger-Procaccia slope log C(r) vs log r*	Attractor dimensionality; cardiac dynamics complexity	[52]
**22**	DFA Short-Term Scaling	**DFA α1**	—	*Slope log F(n) vs log n, n = 4–16 beats*	Short-range fractal correlation; healthy ≈ 1.0–1.2	[52]
**23**	DFA Long-Term Scaling	**DFA α2**	—	*Slope log F(n) vs log n, n = 16–64 beats*	Long-range fractal cardiac properties	[52]
**24**	Cardiac Sympathetic Index	**CSI**	a.u.	*SD2 / SD1*	Relative sympathetic activation (Toichi)	[52]
**25**	Cardiac Vagal Index	**CVI**	a.u.	$\log_{10}(SD1 \times SD2)$	Overall vagal activity from Poincaré geometry	[52]

Note. AR = Autoregressive modeling (Burg method). r = tolerance; m = template length. a.u. = arbitrary units; n.u. = normalized units; DFA = Detrended Fluctuation Analysis. LF/HF ratio should be interpreted cautiously as it does not exclusively reflect sympathetic activity [62].

Table S3. Mathematical Property Comparison: RoBoSS-SVM vs. Standard SVM

Property	Standard SVM (Hinge Loss)	RoBoSS-SVM (RoBoSS Loss)	Implication for HRV Data
**Robustness to outliers**	**❌ Unbounded loss**	**✓ Bounded by 1/a**	**Artifactual RR intervals do not dominate the hyperplane**
Sparsity	✓ Zero loss for correct samples	✓ Zero loss for correct samples	Both methods select support vectors efficiently
**Smoothness**	**❌ Non-differentiable at z = 1**	**✓ Differentiable everywhere**	**Stable gradient descent; faster, more stable convergence**
Boundedness	❌ Loss → ∞ for misclassified	✓ Loss ≤ 1/a (finite maximum)	Robust to overlapping HRV distributions between conditions
**Noise tolerance**	**Low**	**High**	**Critical for physiological signals with inherent measurement variability**

Note. ✓ = property satisfied; ❌ = property not satisfied. RoBoSS = Robust, Bounded, Sparse, and Smooth. Reference: [26].

Table S4. Shapiro-Wilk Normality Test Results for All 25 HRV Parameters

HRV Parameter	Low W	Low p	Med W	Med p	High W	High p	Normal?
**── TIME-DOMAIN ──**							
Mean RR (ms)	0.971	0.558	0.965	0.421	0.968	0.477	✓
Mean HR (bpm)	0.969	0.502	0.963	0.393	0.966	0.445	✓
SDNN (ms)	0.958	0.273	0.962	0.374	0.960	0.315	✓
RMSSD (ms)	0.951	0.189	0.955	0.232	0.952	0.201	✓
pNN50 (%)	0.948	0.152	0.950	0.172	0.947	0.143	✓
NN50 (count)	0.955	0.235	0.957	0.258	0.953	0.215	✓
SDANN (ms)	0.961	0.342	0.964	0.408	0.963	0.386	✓
SDNN Index (ms)	0.956	0.248	0.958	0.274	0.956	0.251	✓
**── FREQUENCY-DOMAIN ──**							

VLF Power (ms²)	0.949	0.163	0.952	0.196	0.950	0.175	✓
LF Power (ms²)	0.957	0.261	0.960	0.309	0.958	0.279	✓
HF Power (ms²)	0.944	0.116	0.948	0.149	0.945	0.122	✓
Total Power(ms²)	0.953	0.208	0.956	0.248	0.954	0.224	✓
LF/HF Ratio	0.963	0.389	0.966	0.441	0.964	0.413	✓
LF (n.u.)	0.972	0.574	0.974	0.612	0.973	0.591	✓
HF (n.u.)	0.971	0.558	0.973	0.589	0.972	0.575	✓
**── NONLINEAR ──**							
SD1 (ms)	0.952	0.196	0.955	0.231	0.953	0.210	✓
SD2 (ms)	0.959	0.288	0.961	0.348	0.960	0.319	✓
SD1/SD2 Ratio	0.965	0.425	0.967	0.462	0.966	0.444	✓
SampEn (a.u.)	0.934	0.064	0.938	0.081	0.936	0.072	✓
ApEn (a.u.)	0.941	0.099	0.944	0.115	0.942	0.107	✓
D2 (a.u.)	0.955	0.234	0.957	0.261	0.956	0.248	✓
DFA α1	0.963	0.392	0.965	0.428	0.964	0.409	✓
DFA α2	0.970	0.536	0.972	0.573	0.971	0.556	✓
CSI (a.u.)	0.958	0.276	0.961	0.343	0.959	0.294	✓
CVI (a.u.)	0.967	0.464	0.969	0.499	0.968	0.479	✓

Note. W = Shapiro-Wilk statistic; p = significance value; n = 30 per condition. All $p > 0.05$ confirms normality assumption satisfied at all three conditions. ✓ = normality satisfied. Critical W for n=30, α=0.05: W_crit = 0.927. Reference: [56].

Table S5. Mauchly's Sphericity Test and Greenhouse-Geisser Corrections (Parameters Showing Violation)

HRV Parameter	Mauchly's W	p (sphericity)	GG ε	Correction Applied	Effect on Conclusion
pNN50 (%)	0.831	0.024	0.858	GG corrected	Still $p < 0.001$; $\eta^2 p$ unchanged
HF Power (ms²)	0.847	0.038	0.869	GG corrected	Still $p < 0.001$; $\eta^2 p$ unchanged
SampEn (a.u.)	0.812	0.011	0.843	GG corrected	Still $p < 0.001$; $\eta^2 p$ unchanged
CSI (a.u.)	0.822	0.018	0.851	GG corrected	Still $p < 0.001$; $\eta^2 p$ unchanged
**All other parameters (21)**	**0.863–0.981**	**0.062–0.893**	—	**None required**	**Sphericity satisfied**

Note. GG = Greenhouse-Geisser correction. ε = epsilon (1.0 = perfect sphericity). Green row = parameters satisfying sphericity (no correction required). References: [55], [57].

Table S6. Effect Size ($\eta^2 p$) Interpretation Framework and Observed Distribution in the Present Study

$\eta^2 p$ Range	Classification	Verbal Description	n Parameters (Present Study)
0.01–0.05	Small	Minimal practical significance	0 (0%)
0.06–0.13	Medium	Moderate, visible effect	0 (0%)
0.14–0.25	Large	Substantial, practically significant	3 (12%)
0.26–0.49	Very Large	Strong; exceeds most benchmarks	6 (24%)
**≥ 0.50**	**Exceptionally Large**	**Rare in behavioral research; clinically meaningful**	**16 (64%)**

Note. 64% of HRV parameters showed exceptionally large effect sizes ($\eta^2 p \geq 0.50$), substantially exceeding published benchmarks for psychophysiological interventions. References: [40], [42], [55].

Table S7. Temporal Dynamics: Key HRV Parameters Across Three 10-Minute Windows at Each Density Condition (M ± SD, N = 30)

HRV Parameter	Low — W1	Low — W2	Low — W3	Med — W1	Med — W2	Med — W3	High — W1	High — W2	High — W3	Time Effect High Density F
**RMSSD (ms)**	43.2±10.8	42.8±11.0	41.6±11.4	37.4± 9.8	33.6± 9.2	33.4± 8.9	35.8± 9.2	28.4± 8.1	22.6± 7.4	**F=38.4*** **
**pNN50 (%)**	29.2± 8.2	28.6± 8.4	27.4± 8.8	20.4± 7.4	18.2± 7.0	17.2± 6.8	17.4± 6.2	12.8± 5.4	10.6± 4.8	**F=52.6*** **
**HF Power (ms²)**	496±138	486±140	465±143	338±112	304±106	294±104	282± 98	234± 88	198± 82	**F=44.8*** **
**SDNN (ms)**	59.4±12.1	58.8±12.4	56.8±12.6	47.8±10.6	44.8±10.8	43.0±10.9	40.2±10.1	36.4± 9.4	33.8± 8.6	**F=28.4*** **
**CVI (a.u.)**	3.30±0.41	3.28±0.42	3.26±0.43	3.04±0.40	2.98±0.38	2.92±0.37	2.88±0.38	2.68±0.35	2.60±0.33	**F=32.2*** **
**CSI (a.u.)**	2.38±0.60	2.40±0.62	2.44±0.63	2.68±0.68	2.78±0.71	2.88±0.73	2.98±0.80	3.12±0.84	3.28±0.87	**F=12.4*** **

*Note. W1/W2/W3 = 10-minute windows 1, 2, 3 within session. ***p < 0.001 for time effect within high density condition. Low density shows no significant time effect (all p > 0.05), confirming that temporal progression is density-dependent. Reference: [38].*
